# Population-representative study reveals cardiovascular and metabolic disease biomarkers associated with misaligned sleep schedules

**DOI:** 10.1093/sleep/zsad037

**Published:** 2023-02-24

**Authors:** Martin Sládek, Jan Klusáček, Dana Hamplová, Alena Sumová

**Affiliations:** Institute of Physiology, The Czech Academy of Sciences, Prague, Czech Republic; Institute of Sociology, The Czech Academy of Sciences, Prague, Czech Republic; Institute of Sociology, The Czech Academy of Sciences, Prague, Czech Republic; Institute of Physiology, The Czech Academy of Sciences, Prague, Czech Republic

**Keywords:** Social jetlag, Chronotype, Circadian Rhythm, Biomarkers, Cholesterol, Lipoproteins, HDL, Lipoproteins, LDL, Models, Statistical, Humans

## Abstract

**Study Objectives:**

Social jetlag manifests as a difference in sleep timing on workdays and free days. Social jetlag is often associated with shorter, lower-quality sleep, so it is unclear how much the chronic circadian misalignment contributes to observed negative health outcomes. We aimed to (1) investigate associations between social jetlag, chronotype (one of its determinants), and the levels of health markers, (2) describe factors associated with social jetlag, and (3) examine whether working from home can reduce social jetlag.

**Methods:**

Adult respondents participated in a nationally representative longitudinal survey of Czech households (individuals in each wave: *n*_2018/19/20_ = 5132/1957/1533), which included Munich ChronoType Questionnaire to evaluate chronotype and social jetlag. A subset provided blood samples (*n*_2019_ = 1957) for detection of nine biomarkers and was surveyed in three successive years (social jetlag calculated for *n*_2018/19/20_ = 3930/1601/1237). Data were analyzed by nonparametric univariate tests and mixed effects multivariate regression with social jetlag, chronotype, sex, age, body-mass index, and reported diseases as predictors and biomarker levels as outcomes.

**Results:**

Higher social jetlag (≥0.65 h) was significantly associated with increased levels of total cholesterol and low-density lipoprotein cholesterol, particularly in participants older than 50 years (Mann–Whitney, men: *p*_CHL_ = 0.0005, *p*_LDL_ = 0.0009; women: *p*_CHL_ = 0.0079, *p*_LDL_ = 0.0068). Extreme chronotypes were associated with cardiovascular disease risk markers regardless of social jetlag (Kruskal–Wallis, *p* < 0.0001). Commuting to work and time stress were identified as important contributors to social jetlag. Individual longitudinal data showed that working from home decreased social jetlag and prolonged sleep.

**Conclusions:**

We report significant associations between sleep phase preference, social jetlag, and cardio-metabolic biomarkers.

## Graphical Abstract

**Graphical Abstract:**
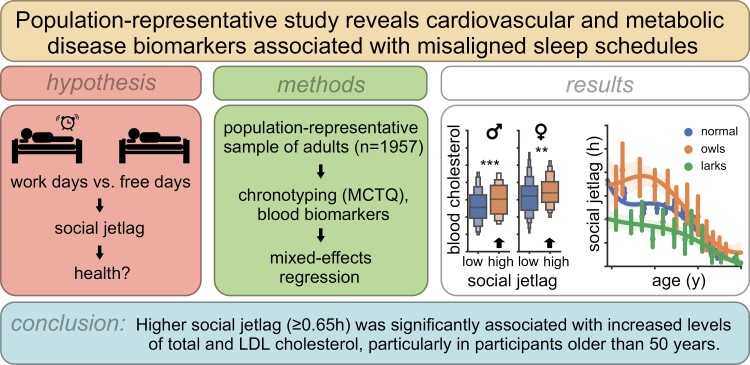

**Statement of Significance**

Social jetlag, or chronic misalignment between biological and social time, is the most common disturbance of circadian rhythms in modern society. It remains unclear, however, whether social jetlag has any negative health effects. By investigating a unique population-representative dataset, including 1957 blood samples analyzed for nine biomarkers, we report significant associations between sleep phase preference, social jetlag, and cardio-metabolic biomarkers. We identify novel factors affecting social jetlag and demonstrate that flexible working schedule efficiently alleviates social jetlag.

## Introduction

Circadian timekeeping serves an essential regulatory function that is common for most organisms, including humans and governs a wide spectrum of physiological, biochemical, or behavioral processes [1]. In mammals, the timekeeping system is hierarchically organized and governed by an endogenous clock capable of producing rhythms in absence of any regular external cues (Zeitgebers) with approximately 24-hour (circadian) period, ranging from cellular activity to complex behavior, despite being very sensitive to many of them, chiefly to the light–dark cycle [2, 3]. In humans, the circadian system evolved to promote sleep during the solar night and wakefulness during the day, giving rise to many associated physiological rhythms such as body temperature and hormone release. When the rhythms are disturbed, be it through internal (such as disintegration of rhythms during aging) or external factors (such as misalignment between circadian clock and light–dark cycle caused by shift work), both physical [4, 5] and mental [6] health can be negatively affected.

Circadian clocks can differ between individuals due to a range of factors, including genetics, age, developmental history, geographic location, and social environment [7]. These differences arise in the preference for a distinct timing of sleep and activity over 24 hours, which often reflects in their schedules relative to the solar time [8]. We refer to this preference as chronotype [9] and it is nowadays considered a complex trait that mirrors circadian clock’s phase in conditions free of social constraints [10]. It is possible to determine the phase of the clock accurately by analyzing the dim light melatonin onset [11], expression of clock genes [12]. Phase can be also estimated by less invasive methods such as actigraphy, core body temperature measurement, or using validated questionnaires such as Morningness–Eveningness Questionnaire (MEQ) [8] and Munich ChronoType Questionnaire (MCTQ) [10]. MEQ is a measure of an individual’s diurnal preference, or their reported inclination for engaging in various activities during the morning or evening. This preference may or may not correspond to the individual’s actual behavior and lifestyle patterns. On the other hand, MCTQ is a measure of an individual’s self-reported sleep timing. Instead of reporting their general preferences for morning or evening activities, it describes individual’s actual behavior. Therefore, while the MEQ assesses a subjective diurnal preference [13], the MCTQ focuses on their objective lifestyle patterns [10]. These two (subjective and objective) measures of chronotype are distinct from each other and provide different information about an individual’s circadian rhythms and lifestyle patterns. Many reports suggest that late chronotypes are associated with increased smoking [10], drinking alcohol [10], depressive symptoms [14, 15], or cardio-metabolic disease markers [16, 17], possibly due to their increased susceptibility to circadian disturbance [10].

One of the most common disturbances of the timekeeping system [18] results from a chronic misalignment between the biological time (determined by chronotype) and external pressures such as a need to use alarm clock to align sleep/wake cycle with social time. We call the phenomenon social jetlag and it can be quantified using MCTQ as a shift between activity–sleep phase during workdays and free days [19]. Major circadian misalignment typically arises from shift work that around 20% of workers worldwide experience [20]. In contrast, social jetlag is perceived by more than 80% of workers because it involves also those working regular hours, which, however, are not aligned with their chronotype [21]. Social jetlag is much more prevalent in adolescents and with late chronotypes and less common in elderly and those with early chronotypes, though neither relationship is linear [10]. Although misalignment due to shift work is larger, social jetlag in regular-hour workers usually persists for a much longer period of their life [19]. Consequently, they experience shortening of sleep duration, causing long-term sleep deprivation and suboptimal timing of physical activity during the working days. Moreover, social jetlag may be associated with additional negative health and socioeconomic outcomes. While the causal relationship is unknown, social jetlag is associated with harmful behavior (smoking, drinking alcohol) [22], lower academic performance [23], lower physical activity [24] and at least in some cohorts, higher body-mass index [25, 26]. While social jetlag compensates for sleep debt usually accumulated during workdays [10], the circadian misalignment it causes may represent a significant health factor [27]. Indeed, recent epidemiological studies have suggested that social jetlag is a risk factor for metabolic syndrome and diabetes [28–30], cardiovascular disease [31–33], or depression [34, 35]. Previous studies also reported that social jetlag affects a number of endocrines [36] metabolic [31, 37], and immune markers [26, 38].

However, such data are often obtained from cross-sectional studies focused on a narrowly defined cohort, or lack detailed descriptions of their samples [39]. Moreover, many factors that can influence social jetlag have previously not been taken into account during statistical analyses and number of biomarkers analyzed in the same cohort is often limited. As a result, the actual health outcomes of social jetlag are still controversial and not well defined. Importantly, due to their multifactorial nature, social jetlag could not be assigned as their definite cause.

We hypothesized that nationally representative, interdisciplinary, and longitudinal household survey with a broad scope may uncover previously overlooked factors contributing to social jetlag, and, in combination with biomarker profiling, it would allow us to reveal unambiguous link between the circadian misalignment and health outcomes. Our primary (aim 1) was to investigate the association of social jetlag and chronotype (one of its determinants), with the levels of nine blood-derived biomarkers related to metabolism, cardiovascular, endocrine, and immune systems. Our secondary aims took advantage of the broad interdisciplinary scope of the survey to find novel determinants of social jetlag (aim 2) and of the longitudinal aspect of the survey to compare the effect of working from home on the individual’s social jetlag (aim 3). We used dimensionality reduction techniques, detailed visualization, and multivariate statistical models. Our study identified cardiovascular and metabolic biomarkers associated with both social jetlag and extreme chronotypes and present a case for including more flexible working schedules to improve workers’ health.

## Methods

### Ethical considerations

The study followed the principles of the Declaration of Helsinki and was approved by Ethics Committee of the Institute for Clinical and Experimental Medicine in Prague. Written informed consent was obtained from each participant who provided blood samples prior to enrollment in the study after explanation of the study procedures.

### Study participants and data collection (aims 1–3)

**Figure 1.**
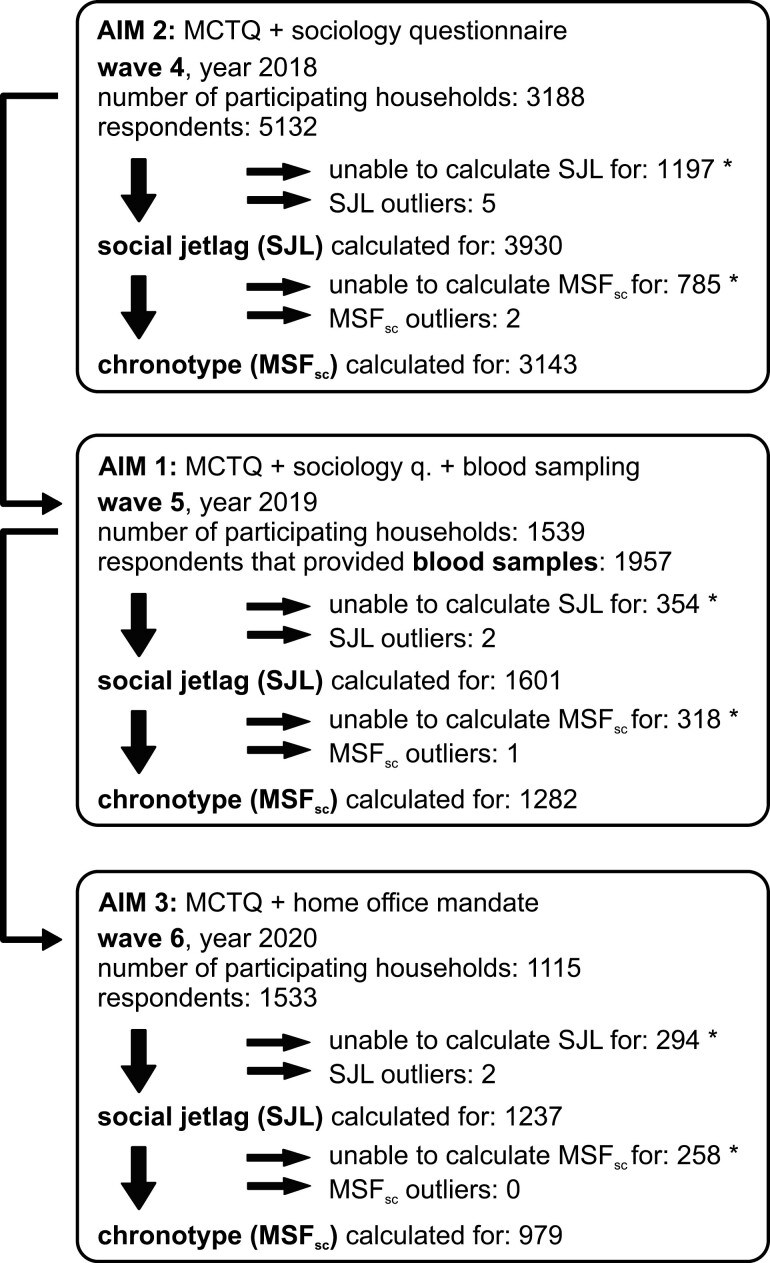
Flowchart of the population during subsequent waves. It shows the number of households that participated in the survey, all individual adult respondents that filled out the MCTQ and all individuals with successfully calculated value of their social jetlag (SJL) and chronotype (MSF_sc_); * denotes irregular sleepers or those that were otherwise unable to provide accurate or correct assessment of their schedule; a small number of outliers >5 standard deviations were filtered out. Minor loss of data occurred also during collection of other variables (e.g. during measurement of LDL cholesterol), resulting in slightly lower n in models that included more variables (for exact number of observations in each model, see Supplementary Tables S2–8). See Supplementary Table S1 for descriptive statistics of all variables.

Czech Household Panel Survey (DOI: 10.14473/ Czech Household Panel Survey 401–402) was a nationally representative longitudinal survey of the Czech noninstitutionalized individuals living in private households (conducted by: Institute of Sociology, Czech Academy of Sciences; CERGE-EI; Faculty of Social Studies, Masaryk University). The households were selected by a two-stage stratified probability sampling design; questionnaires were administered through face-to-face and pen-and-paper-personal interviews. The fieldwork was organized by MEDIAN and STEM/MARK agencies in six subsequent waves (from 2015 to 2020). For the study, only data from waves 4 to 6 were used, which included the Czech language translation of MCTQ [9, 40]. In wave 4, 5132 individual respondents in 3188 households were interviewed (between June and October 2018, data for aim 2). A subsample of the original households was invited to participate in the specialized wave 5 which included blood samples collection between July and November 2019 (aims 1, 3). A further subsample of households was then interviewed during wave 6 in 2020 between May and July; this was during the early stages of coronavirus disease 2019 pandemic, when emergency social distancing laws were enacted in Czechia (aim 3). See the flowchart in Figure 1 for exact number of participants and retention rates in each wave of the survey. The MCTQ questionnaires were administered only to adult participants (*n* = 1–6/household, age = 18–97 years).

### Blood samples (aim 1)

During wave 5, participants were asked to visit a regional medical facility linked to a certified diagnostic company and provide a morning blood sample while fasting (*n* = 1957); the time of sampling was 8.32 ± 1.13 hours (AM, mean ± standard deviation). Plasma levels of high- and low-density lipoprotein cholesterol (HDL, LDL), total cholesterol (CHL), triglycerides (TAG), glucose, C-reactive protein, cortisol, testosterone and dehydroepiandrosterone sulfate (DHEAS) were measured by standard clinical methods (levels of CHL, LDL, HDL, TAG, and glucose are expressed in mmol/l; C-reactive protein in mg/l; cortisol, testosterone in nmol/l; DHEAS in µmol/l; see Supplementary Table S1 for details). Atherogenic index of plasma (AIP) was calculated as log(TAG/HDL). To minimize batch effects introduced by slightly different sensitivity, accuracy, or precision of analytical instruments of five participating diagnostic companies with eight individual laboratories, biomarker levels were median-centered by subtracting a small correction calculated as the difference between median of measurements in a given lab and the median of all measurements.

### Circadian variables (aims 1–3)

The absolute value of the difference between mid-sleep phase on free days and workdays was defined as social jetlag. Objective chronotype (mid sleep phase - sleep corrected (MSF_sc_)) and subjective midpoint of perceived best alertness (Bamid) were determined as described previously [40]. Bamid is a proxy variable for subjective perception of individual’s chronotype, which asks for the start and end of the participant’s best alertness and activity period and is meant to partially emulate the MEQ questionnaire output without the need for 19 additional questions. Age- and sex-corrected normalized chronotype (MSF_sasc_, a hypothetical chronotype at the age of 30) was calculated by fitting third-degree polynomial curves to MSF_sc_ data for men and for women aged ≥18 and normalizing to age = 30 [40, 41]. As a proxy variable to measure extreme chronotypes, we created an additional variable that quantified the spread from the population median of normalized MSF_sasc_ chronotypes (median absolute deviation, MAD_MSF_sasc_). Small numbers (see Figure 1) of outliers >5 sigmas of variables chronotype, social jetlag, and average sleep duration were filtered out. Variables affecting the circadian system (circadian variables) were calculated separately for each of the three waves. See the flowchart in Figure 1 for exact number of participants with successfully calculated circadian variables in each wave.

### Categorization of variables (aims 1–2)

Participants from the lowest and highest MSF_sasc_ deciles were categorized as “Extreme early” and “Extreme late” chronotypes, respectively, and the remaining eight deciles were categorized as “Non-extreme” chronotypes as previously [40]. Instead of selecting arbitrary cutoffs for continuous and ordinal variables, which would result in unevenly sized populations, we used K-means clustering to divide the participants automatically into two equally sized bins (using quantile strategy of KBinsDiscretizer (scikit-learn Python package) based on wave 4 data: low social jetlag (<0.65 h) and high social jetlag (≥0.65 h); younger (max. age = 50) and older (min. age = 51); lower education (max. education = Apprenticeship with matriculation), and higher education (min. education = Vocational secondary school with matriculation). Remaining variables were categorized manually as needed (see respective figure legends for details).

### Composite variables (aims 1–2)

Body-mass index (BMI) was calculated based on the participants’ self-reported current height and weight during wave 5. “Time stress” was calculated as a composite index of five questions on general feelings of stress and time pressure, need for more regular sleep, more time for himself, for family, and for career development. “Diseases” were calculated as a composite index of 12 questions about the diagnosis of heart and circulatory problems, high blood pressure, breathing problems, allergies, back and cervical pain, muscle and joint pain in the hands, arms, feet or legs, stomach and digestion problems, skin problems, severe headaches, diabetes or cancer, and one general question about the presence or absence of any health problems. The remaining variables are self-explanatory. Formulation of the questions translated to English, coding of responses, variable names, and detailed description of composite variables are included in Supplementary Table S1.

### Cross-sectional univariate analyses and visualizations (aims 1–2)

Data were analyzed using Python pandas and visualized by matplotlib and seaborn. Cross-sectional analyses (aim 1, 2) were performed by Mann–Whitney or Kruskal–Wallis tests (scipy.stats) with Dunn’s post hoc multiple comparison tests (scikit_posthocs) were used to compare groups, with Pearson’s or Spearman’s correlation (scipy.stats) [42] to analyze linear relationships and partial Spearman correlation (pingouin) to analyze the association between variables while controlling for confounding variables. Spearman’s correlation index (rho) between all variables was inspected using Hinton diagram. Relationships between selected variables (aim 2) were additionally visualized by polynomial curves fitted using seaborn’s x_estimator function to calculate means and standard deviations in a set amount of bins (for respective numbers of bins and polynomial degree, see figure legends). Histograms were fitted with a kernel density estimate curve using seaborn.

### Longitudinal analysis (aim 3)

Longitudinal analysis of paired circadian variables calculated in the same participants during 2019 and 2020 was performed by Wilcoxon signed-rank test (scipy.stats).

### Dimensionality reduction techniques (aims 1–2)

Linear discriminant analysis was used for separation between participants with low and high social jetlag based either on social jetlag components (aim 2) or solely on the levels of nine measured biomarkers (aim 1). Separation was visualized by histogram of discriminant function and tested by Mann–Whitney; the allocation accuracy was visualized by confusion matrix. Additionally, t-distributed Stochastic Neighbor Embedding [43] was used to visualize clusters of low and high social jetlag quantiles based on social jetlag components (aim 1). For the list of independent variables used in dimensionality reduction techniques, see table or figure legend. Both linear discriminant analysis and t-distributed Stochastic Neighbor Embedding were implemented by scikit-learn [44].

### Cross-sectional multivariate analyses (aims 1–2)

To model the association biomarkers (aim 1), level of each biomarker was included as outcome in a separate Multiple Linear Regression Mixed Effects model (Supplementary Tables S2A–J), and explanatory variables that were strongly associated with social jetlag were omitted, unless they were suspected to have a significant independent effect on the plasma level of the biomarker (age, sex, sleep quality, income, education, diseases, feeling unhealthy, sport, alcohol amount and frequency, smoking, and fruits and vegetables consumption). We additionally included sampling time among the explanatory variables, together with BMI, that is, strongly associated with various metabolic and inflammatory markers and with the levels of biomarkers that were not tested as the outcome. For cholesterol model, HDL and LDL were removed from the list of exposures to avoid strong multicollinearity due to total cholesterol being a composite of HDL + LDL; similarly, for HDL and LDL, total cholesterol was omitted. Household ID code was the group factor. We then simplified the models of cholesterol (Table 1 and Supplementary Table S3), LDL (Table 1, Supplementary Table S4), HDL (Table 2, Supplementary Table S5), triglycerides (Table 2, Supplementary Table S6), and AIP (Table 2, Supplementary Table S7) by including only the most relevant exposures as covariates. Null models without the main explanatory variable social jetlag (Tables 1, Supplementary Table S3–S4) or mean median deviation of normalized chronotype (MAD_MDF_sasc_, Tables 2, Supplementary Table S5–S7) were tested by Chi-square test against models with those explanatory variables.

**Table 1.** Mixed linear regression model showing association between total (CHL) or LDL cholesterol and social jetlag

Outcome	*Exposure:*	*Social jetlag*	*Chronotype*
*Total cholesterol*	*Coef.*	0.13	−0.025
	*Std.Err.*	0.04	0.028
	*z*	3.287	−0.899
	*p >|z|*	** 0.001	0.369
*LDL cholesterol*	*Coef.*	0.109	−0.018
	*Std.Err.*	0.038	0.027
	*z*	2.88	−0.667
	*p >|z|*	** 0.004	0.505

Social jetlag and MSF_sc_ chronotype were modeled as predictor variables (exposure). Controlled for the following covariates: sex (*p*_CHL_ < 0.0001, *p*_LDL_ = 0.038), age (*p*_CHL_ = 0.003, *p*_LDL_ = 0.27), body-mass index (*p*_CHL_ < 0.0001, *p*_LDL_ = 0.89), index of reported diseases (*p*_CHL_ = 0.021, *p*_LDL_ = 0.042), triglyceride levels (*p*_CHL_ < 0.0001, *p*_LDL_ < 0.0001) and HDL cholesterol levels (*p*_LDL_ = 0.044). Household ID code was included to model the random (group) effect. For full tables with confidence intervals, see Supplementary Tables S3–4. Significant chi-square test *P-*values marked with asterisks.

**Table 2.** Mixed linear regression model showing the association between HDL cholesterol, TAG, or AIP and extreme chronotypes (i.e. median absolute deviation of normalized chronotype, MAD_MSF_sasc_)

Outcome	*Exposure:*	*Social jetlag*	*MAD_MSF*_*sasc*_
*HDL cholesterol*	*Coef.*	0.01	−0.032
	*Std.Err.*	0.013	0.015
	*z*	0.741	−2.176
	*p > |z|*	0.459	* 0.03
*Triglycerides*	*Coef.*	−0.018	0.085
	*Std.Err.*	0.03	0.032
	*z*	−0.603	2.62
	*p > |z|*	0.546	** 0.009
*AIP*	Coef.	−0.021	0.078
	Std.Err.	0.023	0.025
	z	−0.926	3.108
	*p* > |z|	0.354	** 0.002

Social jetlag and MAD_MSF_sasc_ were modeled as predictor variables (exposure). Controlled for the following covariates: sex (*p*_HDL_ < 0.0001, p_TAG_ = 0.19, *p*_AIP_ < 0.0001), age (*p*_HDL_ = 0.012, *p*_TAG_ = 0.79, *p*_AIP_ = 0.67), body-mass index (*p*_HDL_ < 0.0001, *p*_TAG_ < 0.0001, *p*_AIP_ < 0.0001), index of reported diseases (*p*_HDL_ = 0.29, *p*_TAG_ = 0.19, *p*_AIP_ = 0.017), LDL levels (*p*_HDL_ = 0.06, *p*_TAG_ < 0.0001, *p*_AIP_ < 0.0001), triglyceride levels (*p*_HDL_ < 0.0001) or HDL levels (*p*_TAG_ < 0.0001). Household ID code was included to model the random (group) effect. For full table with confidence intervals, see Supplementary Tables S5–7. Significant chi-square test *P-*values marked with asterisks.

To model social jetlag as dependent variable (outcome, aim 2), we calculated Multiple Linear Regression Mixed Effects model (Supplementary Table S8) and variables that were hypothesized to plausibly contribute to or be associated with its size (such as chronotype, age, or sex) were included as fixed and independent (explanatory) effects. Household ID code was used as the group identifier to model random effects and avoid distortion due to nesting of participants within households. No further interaction effects were included in the mixed effects model.

Multiple Linear Regression Mixed Effects model was implemented using stats models package [45]. Supervised machine learning method Random Forest implemented using scikit-learn package was used to classify the relative importance of predictor variables for the social jetlag, with maximum depth of 25 trees and 50 estimators.

### Data availability

Raw data (waves 4–5) and technical information are freely available in Czech Social Science Data Archive [46].

## Results

### Blood samples and social jetlag assessment

**Figure 2.**
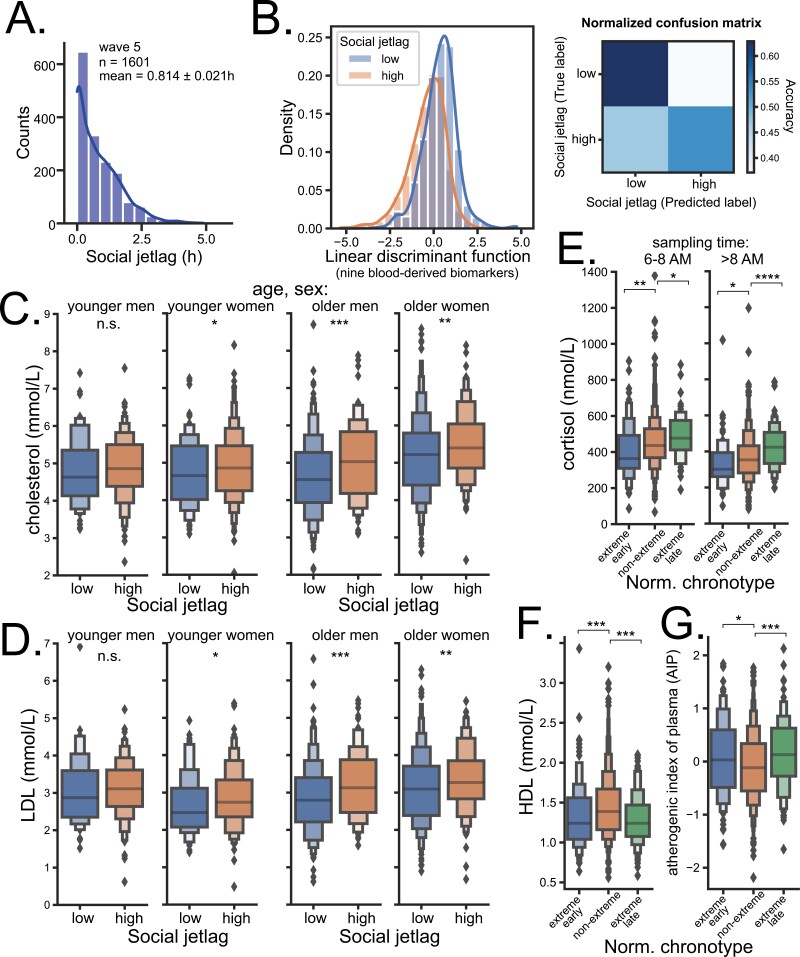
Cardio-metabolic biomarkers are associated with social jetlag and extreme chronotype. (A) Histogram of distribution of social jetlag of participants that provided blood samples during wave 5. (B) Linear discriminant analysis of all analyzed biomarkers (social jetlag group as dependent variable). Histograms show distribution of first linear discriminant function of low and high social jetlag groups (see Methods for categorization details); Mann–Whitney test of the difference between linear discriminant of low and high social jetlag group *p* = 1.14 × 10^−42^. Confusion matrix shows accuracy with which each participant can be allocated to low or high social jetlag group based entirely on their biomarker levels (predicted label) versus their true label; overall accuracy was 0.64 ± 0.03. (C) Total cholesterol levels are significantly (Mann–Whitney test) higher in older men and women with high social jetlag (≥0.65 h) than in those with low social jetlag; this relationship is much less pronounced or missing entirely in younger cohorts. (D) Low-density lipoprotein levels are significantly higher in older men and women with high social jetlag than in those with low social jetlag; this relationship is much less pronounced or missing entirely in younger cohort. (E) Higher cortisol levels are significantly associated (Kruskal–Wallis and Dunn’s post hoc test) with extreme late chronotype (highest decile of normalized chronotype MSF_sasc_), while lower cortisol levels are significantly associated with extreme early chronotypes (lowest decile of MSF_sasc_); the relationship persists regardless of time of sampling. (F) HDL levels are significantly decreased, (G), while AIP is significantly increased in both extreme chronotypes. * *p* < 0.05, ** *p* < 0.01, *** *p* < 0.001, **** *p* < 0.0001.

As the primary aim, we analyzed fasting plasma levels of five cardio-metabolic markers (glucose, total cholesterol, low-/LDL and high-density lipoproteins/HDL, and triglycerides), three hormones (cortisol, testosterone, dehydroepiandrosterone sulfate/DHEAS) and a marker of inflammation (C-reactive protein/CRP). We were unable to assess social jetlag for 354 participants and excluded two more participants as gross outliers (Figure 1), but we successfully quantified social jetlag for 1601 out of the 1957 participants that provided the blood samples (Figure 2A). With the exception of slightly increased glucose (participants with quantified social jetlag vs. participants without social jetlag information, 5.38 ± 1.37 vs. 5.60 ± 1.37 mmol/L, mean ± SD, Mann–Whitney test *p* = 0.0013), the 354 participants with unknown social jetlag did not show any discrepancy in biomarker levels and were not considered in further analyses.

### Biomarker levels differ between low and high social jetlag participants

To investigate biomarkers potentially associated with social jetlag, we divided the participants with quantified social jetlag into low (<0.65 h, cutoff determined by K-means clustering, see methods) and high (≥0.65 h) social jetlag groups. We then performed linear discriminant analysis with all nine measured biomarkers and showed a significant (Mann–Whitney, *p* = 1.14E-42) separation between low and high social jetlag groups, suggesting that social jetlag was in fact associated with at least some of the biomarkers (Figure 2B, left). The overall accuracy of prediction of whether a participant belongs to the low or high social jetlag group based on the biomarker levels was 63.8% (Figure 2B, right).

### Total and LDL cholesterol is associated with higher social jetlag

To identify which biomarkers are associated with social jetlag, we constructed Linear Mixed Effects Models for each biomarker, with a set of covariates detailed in methods, including chronotype and BMI. BMI is linked with metabolic and inflammatory biomarkers and positively associated with social jetlag (partial Spearman correlation adjusted for age and sex, rho = 0.04, *p* = 0.0076). The results of the model for each biomarker are summarized in Supplementary Tables S2A–J. Focusing only on circadian variables revealed the most interesting results for total cholesterol (Supplementary Table S2A) and LDL (Supplementary Table S2B)—both markers showed significant positive association with social jetlag in the model.

To improve the models, we included only those variables as covariates that we deemed biologically most relevant for cholesterol and LDL. The results for cholesterol (0.13 ± 0.04, *p* = 0.001) and for LDL are summarized in Table 1 (0.109 ± 0.038, *p* = 0.004) and show that their levels were positively correlated with social jetlag, together with sex, triglycerides, diseases index, as well as with age and BMI (cholesterol only; for details on the covariates see Supplementary Tables S3–4). We then visualized the results by showing that both total cholesterol (Figure 2C) and LDL levels (Figure 2D) were indeed significantly increased in both men (Mann–Whitney, cholesterol *p* = 0.0005, LDL *p* = 0.0009) and women (cholesterol *p* = 0.0079, LDL *p* = 0.0068) of the older age cohort (>50 years), but not in younger men (cholesterol *p* = 0.2263, LDL *p* = 0.0955); younger women showed similar trend with much less robust association, cholesterol *p* = 0.0472, LDL *p* = 0.0478).

### Cortisol is associated with chronotype

Mixed effects model suggested that several biomarkers were associated with chronotype. Despite a relatively narrow sampling interval (see methods), we were able to detect a highly significant dependence of sampling time on participant’s MSF_sc_ chronotype (Supplementary Figure S1A, Pearson’s correlation *r* = 0.17, *p* < 0.0001). In effect, cortisol levels showed a significant positive correlation with chronotype (Supplementary Figure S1B, *r* = 0.14, *p* < 0.0001), even when adjusted for sampling time, age, sex, BMI, health problems, sleep quality, and social jetlag (partial Spearman correlation, rho = 0.26, *p* < 0.0001). Cortisol levels also remained highly dependent on age- and sex-normalized chronotype MSF_sasc_ even when categorized according to sampling time into two groups (Figure 2E, Kruskal–Wallis, *p* = 0.0007 and *p* < 0.0001 for before and after 8 am sampling time, respectively), demonstrating a strong association between circadian preference and rhythmically released acute cortisol.

We discuss the glucose and C-reactive protein results in Supplementary Material. Interestingly, the model also suggested that, unlike LDL, HDL cholesterol was not associated with social jetlag but rather with MSF_sc_ chronotype (Supplementary Table S2C, *p* = 0.031). While partial Spearman correlation disputed the result of mixed effects model (rho = −0.003, *p* = 0.93), we explored the relationship further in light of our previous results.

### HDL, triglycerides, and atherogenic index are associated with extreme chronotypes

Our previous publication on a different dataset of adult participants from two Czech townships revealed that HDL is significantly lower in both extreme early and late age- and sex-normalized MSF_sasc_ chronotypes [40], a result that we have now confirmed using this independent, larger, and more representative dataset (Figure 2F, Kruskal–Wallis, *p* < 0.0001). Moreover, AIP, a cardiovascular disease risk predictor [47], was significantly increased in both extreme MSF_sasc_ chronotypes (Figure 2G, Kruskal–Wallis, *p* = 0.0001). To explore further, we calculated mixed effects models where instead of chronotype we included the spread from the median of chronotypes (median absolute deviation, MAD_MSF_sasc_, see methods) as the explanatory variable. While the cardioprotective HDL fraction of cholesterol was significantly negatively correlated with MAD_MSF_sasc_ (Table 2, −0.032 ± 0.015, *p* = 0.03), both the level of triglycerides (Table 2, 0.085 ± 0.032, *p* = 0.009) and atherogenic index (Table 2, 0.078 ± 0.025, *p* = 0.002) were significantly positively correlated with MAD_MSF_sasc_, implying increased risk of developing cardiovascular diseases in extreme chronotypes regardless of social jetlag (for details on the covariates see Supplementary Tables S5–7). Importantly, sleep duration was not a confounding variable, as its inclusion in the model did not diminish the association of extreme chronotypes with HDL, triglycerides, and atherogenic index meaningfully. However, HDL also showed significant negative association with sleep duration (−0.028 ± 0.009, *p* = 0.001). Our overall results as well as a previously published body of evidence strongly suggests that both social jetlag and chronotype are important health factors.

### Social jetlag components

**Figure 3.**
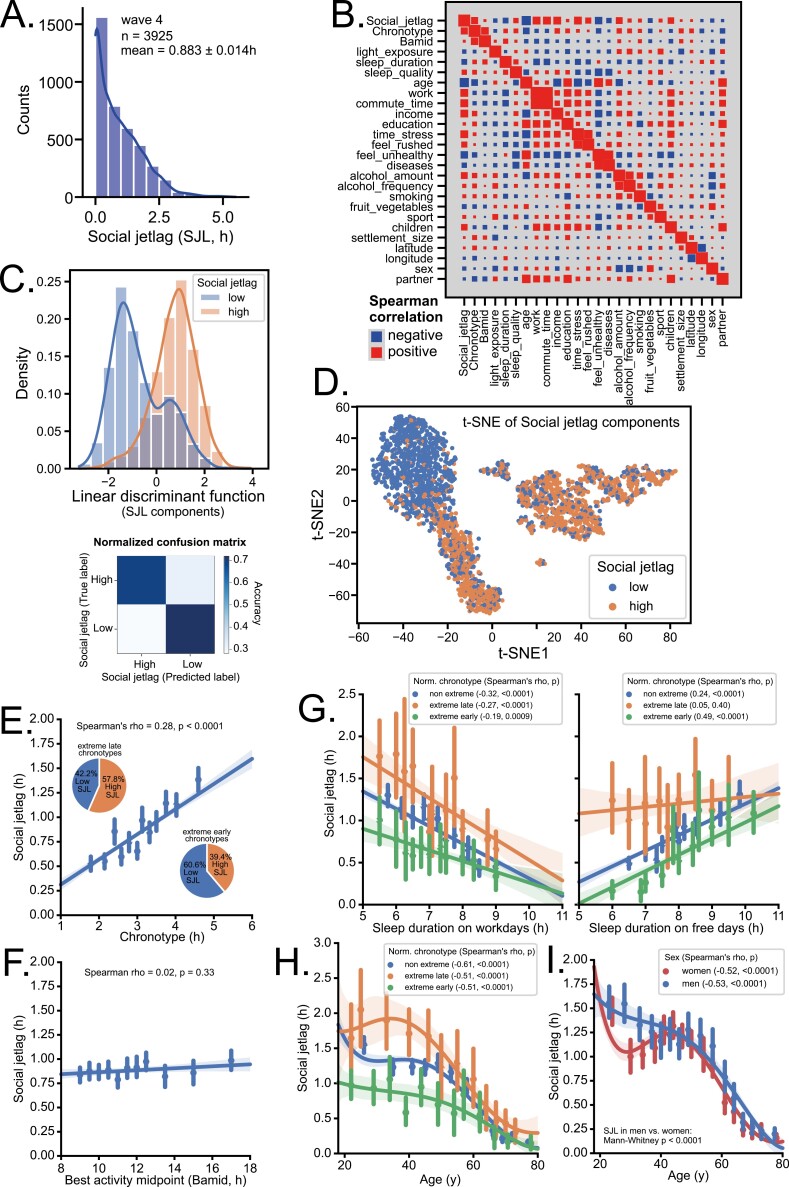
Social jetlag and its components. (A) Histogram of distribution of social jetlag in the Czech population. (B) Hinton diagram of correlations between social jetlag and its components. Size of the square is proportional to Spearman’s rho. For detailed description of variables, see Supplementary Table S1. (C) Linear discriminant analysis of all social jetlag components shown in (B, social jetlag group as dependent variable). Histograms show distribution of first linear discriminant function of low and high social jetlag groups (see Methods for categorization details); Mann–Whitney test of difference between linear discriminant of low and high social jetlag group *p* = 1.16 × 10^−213^. Confusion matrix shows accuracy with which each participant can be allocated to low or high social jetlag group (predicted label) vs. their true label; overall accuracy was 0.774 ± 0.02. (D) *t*-distributed Stochastic Neighbor Embedding of all social jetlag components shown in (B, social jetlag itself excluded) labeled according to social jetlag group shows separation of low and high social jetlag individuals. (E) Social jetlag correlates positively with chronotype (MSF_sc_). The pie charts show different proportions of low and high social jetlag participants within extreme chronotypes (lowest and highest deciles of normalized chronotype MSF_sasc_). (F) Social jetlag does not correlate with self-reported midpoint of best activity and highest alertness (Bamid). (G) Social jetlag correlates negatively with sleep duration on workdays (left) and positively with sleep duration on free days (right). Social jetlag correlates negatively with age for all chronotypes (H) and both sexes (I). Spearman’s correlation; for visualization purposes, data were separated into 12 bins with mean ± SD error bars; for age correlations, fifth order polynomial was used to better visualize the relationship.

In our previous publication, we explored the factors associated with chronotype [40]. To examine the factors that are associated with social jetlag, we took advantage of our largest dataset collected without blood sampling (Figure 1). The social jetlag distribution during wave 4 is shown in Figure 3A, see Supplement Material for more details. We plotted regression indexes of all analyzed variables (Figure 3B). By inspecting the first line or column, it is clear that apart from directly related circadian factors such as chronotype and average sleep duration, social jetlag was strongly correlated with age, weekly time spent in all jobs (work), weekly time spent commuting to work (commute time), income, time stress (composite index of five questions about time pressure) and others. Linear Mixed Effects Model (Supplementary Table S8, see methods for details) revealed that social jetlag was highly significantly associated with chronotype, age, education, time stress, sleep duration, subjective chronotype (Bamid), work, sleep quality, daylight exposure, and household income. For more detailed description and Random Forest ranking of variables, see Supplementary Material.

To verify the explanatory variables as components of social jetlag, we performed linear discriminant analysis with all the independent variables and compared the linear discriminant function between low and high social jetlag groups (Figure 3C), showing highly significant separation between them (Mann–Whitney, *p* = 1.16E–213). The overall accuracy of prediction of whether a participant belongs to the low or high social jetlag group based on the explanatory variables (social jetlag components) was 77.4%. Furthermore, we performed t-distributed Stochastic Neighbor Embedding of all social jetlag components and showed that the low social jetlag group cluster is visibly separated from the high social jetlag cluster (Figure 3D).

### Circadian components, age, and sex

Social jetlag positively correlated with MSF_sc_ chronotype as well as with normalized chronotype (MSF_sasc_), with 61% of extreme early chronotypes but only 42% of extreme late chronotypes showing low social jetlag (Figure 3E). The correlation remained significant even after adjusting for age, sex, and Bamid (partial Spearman correlation rho = 0.2, *p* < 0.0001). However, social jetlag did not correlate with Bamid (Figure 3F), unless adjusted for “objective” MSF_sc_ chronotype (partial Spearman correlation rho = −0.11, *p* < 0.0001). Social jetlag negatively correlated with sleep duration on workdays (Figure 3G, left)—the later the chronotype, the steeper the regression line, and the more significant the correlation (see Figure 3G for Spearman’s rho, *p*). The opposite was true for positive correlation of social jetlag with sleep duration on free days, as it disappeared completely in late chronotypes but it was the most pronounced in early chronotypes (Figure 3G, right). Social jetlag was highly dependent on age, regardless of chronotype (Figure 3H), or sex (Figure 3I). There was a small difference between the average social jetlag in men (0.95 ± 0.02 h) and women (0.83 ± 0.02 h, Mann–Whitney, *p* < 0.0001, Figure 3I); however, after adjusting for other factors using the mixed effects model, any difference between social jetlag of men and women disappeared (Supplementary Table S8). Moreover, random forest ranked sex as the least important of all tested social jetlag explanatory variables (see details in Supplementary Material).

### Social components of social jetlag

**Figure 4.**
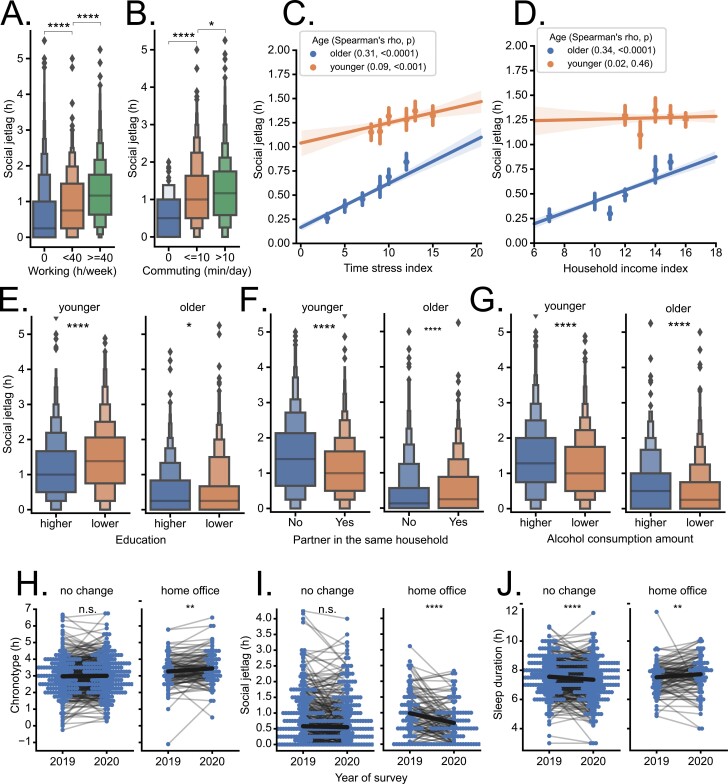
Social components and benefits of flexible working schedule. (A) Participants working 40 or more hours/week have significantly higher social jetlag (Kruskal–Wallis and Dunn’s post hoc test) than those working less or not at all. (B) Commuting to work is associated with higher social jetlag (only workers were analyzed). (C) Social jetlag correlates positively with feelings of time stress (composite index of five questions about time pressure, regular sleep, and time for self, family, and career; for details, see Supplementary Table S1), mean ± SD, six bins. (D) Social jetlag correlates positively with household income in older (<50 years old) but not in younger (≤50 years old) participants, six bins. (E) Younger participants (left) with higher education (see Methods for categorization, Mann–Whitney test) have significantly lower social jetlag than those with lower education, while this trend is reversed in older participants (right). (F) Younger participants (left) without a spouse, or registered or permanent partner in the same household have significantly higher social jetlag than those with a partner, while this trend is reversed in older participants (right). (G) Higher consumption of alcohol (i.e. those that drink three or more standard drinks in one session) is associated with higher social jetlag in both age cohorts. Pairwise comparison by Wilcoxon test between respective circadian variables analyzed in 2 consecutive years in the same participants that either experienced home–office in 2020 or had no change of working pattern in 2020 shows that working from home significantly increased their chronotype (H), decreased their social jetlag (I) and allowed them to prolong their average weekly sleep duration (J); bold black line connects population means and thin lines connect individual values of chronotype, social jetlag or sleep duration in 2019 and 2020. * *p* < 0.05, ** *p* < 0.01, *** *p* < 0.001, **** *p* < 0.0001

Several variables related to social, economic, and lifestyle factors were highly associated with social jetlag. Unsurprisingly, social jetlag was much lower in participants that were not working (including retirees) than in those working standard 40-hour working week or longer (Figure 4A, Dunn’s test, *p* < 0.0001). Moreover, workers commuting to their place of employment for even a short time interval showed significantly higher social jetlag than workers that did not need to commute (Figure 4B, Dunn’s test, *p* < 0.0001). Interestingly, even very short commutes to place of work further increase social jetlag of workers by more than 30 minutes. Longer commutes increase social jetlag further, but only by additional 4 minutes. Time stress (see Methods for details) was one of the factors most significantly associated with higher social jetlag (age-corrected partial Spearman correlation rho = 0.18, *p* < 0.0001); however, this association was much less pronounced in younger (≤50 years, see methods for categorization details) age cohort (see Figure 4C, D for correlation in age cohorts). Similarly, while social jetlag was highly positively correlated with income in older cohort, this association was not significant in younger cohort (Figure 4D, age-corrected partial Spearman correlation rho = 0.15, *p* < 0.0001). Lower social jetlag was associated with higher education in younger cohort (Figure 4E, left, Mann–Whitney test, *p* < 0.0001), but with lower education in older cohort (Figure 4E, right, *p* = 0.03). Similarly, participants living with spouses or permanent partners had significantly lower social jetlag in younger cohort (Figure 4F, left, Mann–Whitney test, *p* < 0.0001), but higher social jetlag in older cohort (Figure 4F, right, *p* < 0.0001). Finally, unlike the frequency of alcohol consumption (Supplementary Table S8), amount of consumed alcohol was highly positively correlated with social jetlag in both age cohorts (Figure 4G, *p* < 0.0001). Categorizing the participants according to their worker status instead of age yields analogous regression results, with workers similar to younger and nonworkers (and retirees) similar to older group (plots not shown).

### Circadian variables improve in response to changes in the working schedule

We hypothesized that more flexible working schedule that would not require daily commuting to work might lead to improvement in circadian variables. To test the possibility on a scale of representative population, we took advantage of the fact that during the spring of 2020, Czech government-mandated social distancing and working from home (“home office”) for many professions and compared the main circadian variables between 2019 (wave 5) and 2020 (wave 6) in the same participants by pairwise Wilcoxon signed-rank test as part of the aim 3. While there was no difference in MSF_sc_ chronotype (Figure 4H, left, *p* = 0.39) or social jetlag (Figure 4I, left, *p* = 0.41) and shorter sleep duration (Figure 4J, left, *p* < 0.0001) for participants that remained working on the same schedule in 2020 as in 2019, for participants that were on home–office in 2020 MSF_sc_ chronotype delayed significantly by 11 minutes (Figure 4H, right, *p* = 0.0021), social jetlag decreased significantly by 18.6 minutes (Figure 4I, right, *p* < 0.0001), and sleep duration lengthened significantly by 12.6 minutes (Figure 4J, right, *p* = 0.0026). This was coupled with much more pronounced drop in alarm clock use on workdays by participants at home–office (−21%, vs. −3% of those on the same schedule). The participants in home–office reported a less pronounced decrease in outside light exposure experienced (−1.38 h, vs. −2 h of those on the same schedule), while the self-reported sleep quality did not change significantly between 2019 and 2020 in either group (home–office *p* = 0.68, same schedule *p* = 0.0518). The slight shift to later chronotype and decrease in alarm clock use indicates a better alignment of activity/rest schedules with biological time. The longer sleep duration and decreased social jetlag may provide basis for better health outcomes, especially in light of the demonstrated significant correlation between social jetlag and cholesterol levels.

## Discussion

The study builds on our previous report on chronotype distribution in the Czech population [40]. Primary aim of this study was to identify biomarkers associated with social jetlag. We showed that higher cholesterol and LDL levels are significantly associated with higher social jetlag in participants over the age of 50 years. Additionally, we showed that lower HDL and higher atherogenic index are associated with extreme chronotypes. As a secondary aim, we explored novel socioeconomic factors associated with social jetlag. Finally, we also demonstrated in the same participants that change from standard to home–office working schedule (which omits commuting and allows more freedom in setting working hours) markedly decreases social jetlag and increases overall sleep duration, which may provide long-term health benefits.

The main result of the study is the identification of a strong correlation between selected metabolic biomarkers and social jetlag. Using multivariate models, we found significant association of social jetlag with blood cholesterol levels. Since BMI is in our dataset positively associated both with total cholesterol (Table 1) as well as with social jetlag, and obesity, in general, is a factor previously reported to be linked with higher social jetlag [25, 26], it is not surprising to find higher cholesterol levels in participants with higher social jetlag. Indeed, several studies have reported association of social jetlag with cholesterol in different cohorts including preadolescent children [47], midlife adults [31], and patients with hypertension or diabetes [37, 48]. In contrast, a similarly sized study on an older (>50 y) population [28] did not detect any significant association between social jetlag and cholesterol or triglycerides, but reported association of social jetlag with higher glucose levels. Importantly, in our study cholesterol is significantly increased in participants older than 50 years with social jetlag above approximately 40 min, regardless of their sex, indicating there may be certain thresholds beyond which these associations manifest. Importantly, the association between social jetlag and cholesterol did not meaningfully diminish when adjusted for health-related factors such as smoking, alcohol, sleep quality, sport, and preexisting diseases, suggesting that the relationship is quite robust (Supplementary Table S2A). However, to make causal implications, it would be necessary to perform long-term longitudinal studies that are beyond the scope of our report.

Our novel finding is that while LDL fraction behaved similarly to total cholesterol regarding its association with social jetlag, HDL or triglycerides were not significantly linked with social jetlag at all. Instead, HDL was associated with chronotype; specifically, it was negatively correlated with extreme chronotype when adjusted for sex and age, indicating that both extreme early (“larks”) and extreme late types (“owls”) may have compromised blood levels of cardioprotective lipoproteins. The result is in agreement with our previous report on a different smaller cohort sampled from two Czech townships [40]. This was further paralleled by our finding of reversed association of extreme chronotype with both triglycerides and AIP, which represent a robust biomarker of dyslipidemia, atherosclerosis, coronary syndrome, and metabolic syndrome [49]. Thus, while the cardioprotective marker (HDL) is decreased, the cardiovascular disease risk factors (triglycerides, atherogenic index) are increased in extreme chronotypes. Interestingly, the overall data imply that higher social jetlag is not the sole causal factor responsible for increased cardiovascular risk in late chronotypes [16, 17]. Previous studies suggested that instead of sleep phase misalignment, the interaction between chronotype and sleep length may be behind this effect, as both too short and too long sleep duration in early chronotypes and longer sleep duration in late chronotypes increases the odds of being overweight and having elevated blood pressure [50, 51]. This is partly supported by our results showing that HDL levels, which are lower in participants with higher BMI due to association of obesity with dyslipidemia, are also lower in participants with extreme chronotype and with longer sleep duration.

This relationship between MSF_sc_ chronotype and HDL is also markedly different from the detected association of chronotype with cortisol and glucose levels. Unlike HDL, cortisol [52] and glucose [53] levels in blood oscillate with a circadian period even on constant routine and thus are expected to be closely correlated with sampling time. Likewise, MSF_sc_ chronotype clearly affected the time participants were willing to arrive at the local laboratory and provide fasting blood samples in the morning (Supplementary Figure S1). Consequently, both cortisol and glucose levels significantly correlated with chronotype. Moreover, glucose is also extremely sensitive to meals [54] and can be affected by the timing of dinner on the day before morning sampling [55]. In conclusion, without drawing causal inference, we suggest that our data conclusively point to a strong link between sleep phase preference, social jetlag, and certain cardio-metabolic markers.

We observed that although the average social jetlag across all adult cohorts was <1 hour, its magnitude strongly correlated with age and decreased rapidly and linearly after 50 years of age. For workers, the average social jetlag reached 1.2 hours and just 8% of them reported no social jetlag. This is in line with previous studies using MCTQ toolkit [21, 56]. Interestingly, while both MSF_sc_ chronotype and age-/sex-normalized MSF_sasc_ chronotype positively correlated with social jetlag, the proxy variable for a more subjective perception of individual’s chronotype (Bamid) did not correlate with social jetlag, unless adjusted for MSF_sc_—in that case, the correlation was slightly negative. Bamid is a variable independent of actual sleep–wake schedule and thus represents the approximation of MEQ score, even though the two questions that generate Bamid cannot fully reflect the 19-question MEQ. We have previously validated the variable showing that Bamid positively correlates with MSF_sc_ (*r* = 0.33) and can replicate many of its associated features [40]. While most reports suggest that MEQ evening types are more likely to experience social jetlag than morning types [57–59], others found the opposite relationship [60], which is also in better agreement with our data. The result implies that at least some evening types can synchronize their biological time with social time.

Apart from the circadian factors directly contributing to social jetlag, such as chronotype assessed as both MSF_sc_ and Bamid, sleep duration, and outside light exposure, we identified perception of time stress, sleep quality, education, overall weekly working hours, time spent commuting and household income as significant components of social jetlag. A novel strong predictor of social jetlag identified in our study was an index composed of five questions assessing feelings of time stress, indicating that subjective perception of a lack of time leads to accumulation of sleep debt on workdays that are then compensated through social jetlag. We are not aware of previous studies with comparable qualitative assessments of time stress. However, recent reports showed that higher social jetlag is associated with job-related stress [61] and with anxiety symptoms in adolescents [62]. Other psychosocial factors such as professional or school burnout [63, 64] were previously reported to show either no or negative correlation with social jetlag.

Education and income are commonly used as confounders in other studies of social jetlag [28, 65]. They both positively correlated with each other and with weekly working hours, which directly affect sleep timing on workdays. However, while more time spent working and commuting to work increases social jetlag independently of age, both income, and education show interactions that are more complicated. For example, social jetlag is significantly higher in less educated adults ≤50 years, but lower in those >50 years, reflecting a lower percentage of more educated active workers versus less educated early retirees in this age cohort. Overall education level barely correlated with social jetlag unless adjusted for age and work, and then the correlation was negative. This is in support of previous studies showing that social jetlag can be a negative predictor of academic achievement and cognitive abilities [66]. Interestingly, while our mixed effects model identified geographic (latitude, longitude, and municipality size) and social factors (presence of spouse or permanent partner in the household, amount of consumed alcohol) associated with social jetlag in a manner similar to their effect on chronotype [40], it did not identify as significant several factors that were previously considered to be positively or negatively associated with social jetlags, such as smoking [19] or sports activities [67, 68].

Given that metabolic health is linked with the circadian system, it is tempting to speculate that improving circadian parameters would provide health benefits. There have been interesting attempts to counteract social jetlag by intervention, for example by limiting evening blue light or increasing morning bedroom light [69]. The pandemic presented an opportunity to test whether it is actually feasible to improve the circadian misalignment on a population scale. Since a significant proportion of respondents were mandated to work from home in 2020, we analyzed the changes the stay-at-home scheme (“home office”) enacted on their circadian parameters by comparing them individually with their 2019 parameters using pairwise statistics. The detected within-participants change to later chronotype during social restrictions regardless of work schedule was reported previously [70]. In a large-scale online study across 40 countries [71], the MSF_sc_ chronotype increased by half an hour during restrictions. Authors suggested that this increase was not just due to less outside light exposure, since late types with less morning-light exposure did not delay more than early types. Our smaller dataset precluded similar age- or chronotype-specific clustering; however, we showed that even though the group that worked from home in 2020 had on average already lower outside light exposure and later chronotype in 2019, they experienced a smaller drop in light exposure and larger change to later chronotype in 2020 than the control group. It supports the interpretation by Korman et al. that the chronotype change is not caused solely by decrease in morning-light exposure [71]. Most importantly and in agreement with previous publications [71], we detected lower alarm clock use, highly significant decrease in social jetlag and increase in overall sleep duration. While the self-reported sleep quality did not significantly improve by home–office, we detected a trend suggesting worse sleep in 2020 versus 2019 in the control group, but not in the home–office group. However, the sleep quality was assessed only on a simple four-point scale and the test did not reach statistical significance (see Results). Nevertheless, we interpret the finding as a clear net-positive benefit for health due to relaxed pressure of social time on the circadian system and longer sleep.

As a representative study of the entire Czech noninstitutionalized adult population with individual households as basic sampling unit, our study has a major advantage over many other studies published previously. However, it has also several limitations. To begin with, an observational study can only measure associations, not causations. The nature of self-reporting questionnaire may result in possible misclassification of certain variables. The survey was not originally intended for analysis of circadian system and sleep patterns. Its wide scope prevented the inclusion of additional queries of interest besides MCTQ. On the other hand, wide interdisciplinary scope may result in less bias in data reporting compared to targeted specialized surveys. Notably, we have not analyzed meal timing and caloric composition, caffeine consumption, electronic screen time, exercise, and napping patterns, all of which have previously been suggested to affect sleep timing. Especially delayed timing of higher caloric meals have been previously shown to be associated with higher social jetlag [72] and “eating” jetlag has been proposed as an important predictor of metabolic health [73, 74]. These factors should not be omitted from future comprehensive studies of circadian misalignment. Unfortunately, we were unable to reanalyze the blood samples of the same participants during or after the end of pandemic restrictions to detect potential changes in response to the more relaxed social time pressure and thus identify a causal relationship between circadian parameters and health markers. However, it is very likely that the short-term duration of strictest pandemic restrictions in Czechia (March–June; though the percentage of home–office workers would likely stay higher even after this period) would not be sufficient to influence the blood biomarkers significantly in a way that would be detectable in the presence of many confounders. Nevertheless, we believe our data add novel and valuable insight into the importance of circadian system and suggest that flexible working schedules might be beneficial for individual health and well-being.

## Supplementary Material

zsad037_suppl_Supplementary_Material

zsad037_suppl_Supplementary_Table_S1
