# Supplementary material for: Population-representative study reveals cardiovascular and metabolic disease biomarkers associated with misaligned sleep schedules
Source: Sleep. 2023 Feb 24;46(6):zsad037. doi: 10.1093/sleep/zsad037 (PMC10262187; doi:10.1093/sleep/zsad037)
Supplement: zsad037_suppl_Supplementary_Material [file zsad037_suppl_supplementary_material.docx]

# Population-representative study reveals cardiovascular and metabolic disease biomarkers associated with misaligned sleep schedules

Martin Sládek*^1^, Jan Klusáček^2^, Dana Hamplová^2^ and Alena Sumová^1^

^1^ Institute of Physiology, the Czech Academy of Sciences, Prague, Czech Republic

^2^ Institute of Sociology, the Czech Academy of Sciences, Prague, Czech Republic

* Corresponding author (Martin Sládek, PhD, Vídeňská 1083, Prague, Czechia, tel: +420241062609, email: [Martin.Sladek@fgu.cas.cz](mailto:Martin.Sladek@fgu.cas.cz))

# Supplementary results

***Associations of glucose and C-reactive protein with chronotype***

Fasting glucose showed a significant negative correlation with MSF_sc_ chronotype (Fig. S1C, r = -0.09, p = 0.0014); this was also suggested by the mixed effects model (Table S2E, p = 0.003). However, partial Spearman correlation adjusted for the same confounding factors as in the case of cortisol did not show significant association MSF_sc_ chronotype (rho = -0.03, p = 0.25).

For C-reactive protein (Table S2I), the model suggested a positive correlation with chronotype (p = 0.016); however, partial Spearman correlation adjusted for the same confounding factors as in the case of cortisol and glucose did not show significant association (rho = 0.05, p = 0.11).

***Dubious association of higher cortisol levels with low social jetlag***

### In case of cortisol (Table S2F), the negative association with social jetlag suggested by the mixed effects model was significant but its relevance dubious due to cortisol’s high-amplitude diurnal changes in blood, its protein-binding capacity and sensitivity to distress during sampling [43]; nevertheless, cortisol positively correlated with social jetlag even after controlling for confounders such as sampling time, age, sex, BMI, health problems, sleep quality and MSFsc chronotype (partial Spearman correlation, rho = -0.09, p = 0.0014). Remaining biomarkers did not significantly correlate with social jetlag.

### Assessment of circadian variables

In the cohort of participants assessed in the wave 4, we previously showed that the objective (MSF_sc_) and subjective (Bamid, see methods for details) measures of chronotype positively correlate [36], and the average chronotype assessed by both parameters in wave 5 (MSF_sc_: 3.08 ± 0.03 h, Bamid: 12.02 ± 0.07 h, mean ± SEM) did not differ to the previously published data from wave 4 (MSF_sc_: 3.13 ± 0.02 h, Mann-Whitney test p = 0.93; Bamid: 12.08 ± 0.04 h, p = 0.31). Only 24.2% of all respondents had social jetlag = 0 and 39.6% of respondents had social jetlag ≥ 1h. The distribution during wave 4 is shown in Fig. 2A, with average social jetlag of 0.88 ± 0.01 h (n = 3925). During wave 5, there was a slight decrease of average social jetlag (0.81 ± 0.02 h, n = 1601, p = 0.0138). For the rest of circadian and non-circadian variables description, see Supplemental Table S1. For the components of social jetlag, see Supplemental Table S8.

### Social jetlag components

Social jetlag was also to a lesser extent associated with commute time, amount of consumed alcoholic drinks, presence of subject’s partner and underage children in the same household, settlement size, longitude and latitude. The model revealed no significant difference in social jetlag between men and women and no significant correlation of social jetlag with smoking (P = 0.07), frequency of drinking alcohol or eating fruits and vegetables, sport activities, feeling unhealthy or rushed. Composite index of known diagnosed diseases was not associated with social jetlag. To further classify the relative importance of social jetlag components, we used Random Forest model with all the explanatory variables (r = 0.92, root mean squared error= 0.21), which ranked age (relative importance = 0.323), MSF_sc_ chronotype (0.115) and sleep duration (0.07) as the most important factors, followed by Bamid chronotype (0.047), daylight exposure (0.044), education (0.040), time stress (0.036), longitude (0.034), latitude (0.033), work (0.033), income (0.025), commute time (0.024) and alcohol (0.023), with the rest of the variables less important (< 0.02).

# Supplementary Figure


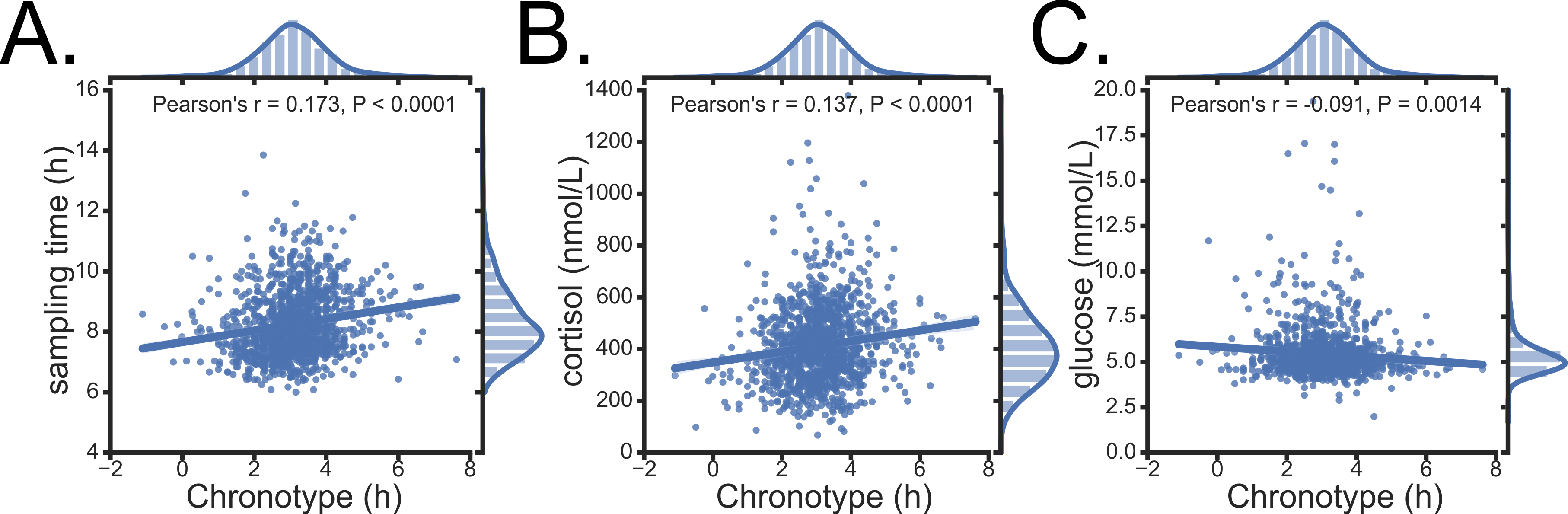


FIG. S1: (A) Subject’s chronotype influenced the time of blood sampling and thus correlated with rhythmically regulated biomarkers, resulting in significant positive correlation of chronotype with level of cortisol (B) and negative correlation with level of glucose (C).

# Supplementary table S1 captions

Tab. S1 (see Supplementary xlsx file S1): Description of variables.

Variable – name of the final analyzed variable; N – number of participants; Mean – arithmetic average; SD – standard deviation; SJL_correlation – Spearman’s correlation index of variable with social jetlag; Dataset – during which waves were variables collected (asterisks mark the dataset quantitatively described in the table); Composite – for variables that are composed of several sub-variables, how was the final variable created; Component_var – name of the sub-variable or abbreviation of the final variable; Value – possible numeric values of the (sub-)variable; Response – possible replies to the question and how they correspond to the specific numeric value; Question – wording of the question about (sub-)variable (English translation).

# Supplementary tables S2-8

Tab. S2A: Mixed effects linear regression model of total cholesterol.

| *Dependent variable:* | ***cholesterol*** | *Log-Likelihood:* | -1440.58 |  |  |  |
| --- | --- | --- | --- | --- | --- | --- |
| *No. Groups:* | 946 | *Model:* | MixedLM |  |  |  |
| *Min. group size:* | 1 | *Scale:* | 0.6532 |  |  |  |
| *Max. group size:* | 3 | *Observations:* | 1094 |  |  |  |
| *Mean group size:* | 1.2 | *Converged:* | Yes |  |  |  |
| ***Variable*** | ***Coef.*** | ***Std.Err.*** | ***z*** | ***P>\|z\|*** | ***CI (0.025)*** | ***CI (0.975)*** |
| ******Social_jetlag** | **0.15** | 0.043 | 3.528 | **< 0.0001** | 0.067 | 0.234 |
| **Chronotype** | **-0.042** | 0.031 | -1.357 | **0.175** | -0.103 | 0.019 |
| ****Sex[T.women] | 0.713 | 0.125 | 5.716 | < 0.0001 | 0.468 | 0.957 |
| **Partner[T.Yes] | 0.208 | 0.074 | 2.794 | 0.005 | 0.062 | 0.354 |
| ****TAG | 0.509 | 0.037 | 13.715 | < 0.0001 | 0.436 | 0.581 |
| **Glucose | -0.075 | 0.024 | -3.058 | 0.002 | -0.122 | -0.027 |
| *Cortisol | 0 | 0 | -2.067 | 0.039 | -0.001 | 0 |
| *DHEAS | 0.03 | 0.013 | 2.282 | 0.022 | 0.004 | 0.055 |
| Testosterone | 0.013 | 0.007 | 1.763 | 0.078 | -0.001 | 0.027 |
| CRP | 0.005 | 0.004 | 1.14 | 0.254 | -0.004 | 0.014 |
| Sleep_quality | 0.03 | 0.045 | 0.656 | 0.512 | -0.059 | 0.118 |
| Sampling_time | 0.008 | 0.028 | 0.267 | 0.789 | -0.048 | 0.063 |
| ****BMI | -0.023 | 0.006 | -3.662 | < 0.0001 | -0.036 | -0.011 |
| **Age | 0.008 | 0.003 | 2.767 | 0.006 | 0.002 | 0.014 |
| Income | 0.002 | 0.013 | 0.128 | 0.898 | -0.024 | 0.027 |
| Education | 0.008 | 0.011 | 0.683 | 0.495 | -0.015 | 0.03 |
| Feel_unhealthy | 0.013 | 0.039 | 0.344 | 0.731 | -0.063 | 0.089 |
| *Diseases | -0.036 | 0.016 | -2.199 | 0.028 | -0.067 | -0.004 |
| Sport | -0.021 | 0.017 | -1.277 | 0.201 | -0.054 | 0.011 |
| Fruits_vegetables | 0.014 | 0.03 | 0.446 | 0.656 | -0.046 | 0.073 |
| Alcohol_amount | 0.02 | 0.02 | 0.957 | 0.338 | -0.02 | 0.06 |
| Alcohol_frequency | 0.002 | 0.02 | 0.08 | 0.936 | -0.037 | 0.04 |
| **Smoking | -0.05 | 0.019 | -2.674 | 0.007 | -0.087 | -0.013 |
| *Children | -0.106 | 0.047 | -2.262 | 0.024 | -0.198 | -0.014 |
| *Intercept* | 4.304 | 0.486 | 8.856 | < 0.0001 | 3.352 | 5.257 |
| Household_ID *Var* | 0.167 | 0.11 |  |  |  |  |

Household code was modeled as a random (group) effect (*Var*), variables affecting biomarker level as fixed effects. For details on the rest of the variables, see Supplemental Table S1. Binary categorical variables (partner, sex) have the reference category designated with T. Asterisks denote the significant associations. * p<0.05, ** p<0.01, *** p<0.001, **** p<0.0001, chi-square test.

Tab. S2B: Mixed effects linear regression model of total low-density lipoprotein (LDL) cholesterol.

| *Dependent variable:* | ***LDL*** | *Log-Likelihood:* | -1383.11 |  |  |  |
| --- | --- | --- | --- | --- | --- | --- |
| *No. Groups:* | 944 | *Model:* | MixedLM |  |  |  |
| *Min. group size:* | 1 | *Scale:* | 0.6484 |  |  |  |
| *Max. group size:* | 3 | *Observations:* | 1091 |  |  |  |
| *Mean group size:* | 1.2 | *Converged:* | Yes |  |  |  |
| ***Variable*** | ***Coef.*** | ***Std.Err.*** | ***z*** | ***P>\|z\|*** | ***CI (0.025)*** | ***CI (0.975)*** |
| ****Social_jetlag** | **0.121** | 0.041 | 2.966 | **0.003** | 0.041 | 0.2 |
| **Chronotype** | **-0.009** | 0.03 | -0.292 | **0.77** | -0.067 | 0.05 |
| **Sex[T.women] | 0.337 | 0.122 | 2.751 | 0.006 | 0.097 | 0.577 |
| *Partner[T.Yes] | 0.145 | 0.071 | 2.039 | 0.041 | 0.006 | 0.284 |
| *HDL | 0.175 | 0.083 | 2.101 | 0.036 | 0.012 | 0.337 |
| ****TAG | 0.334 | 0.037 | 8.963 | < 0.0001 | 0.261 | 0.407 |
| Glucose | -0.043 | 0.023 | -1.828 | 0.068 | -0.088 | 0.003 |
| ****Cortisol | -0.001 | 0 | -3.878 | < 0.0001 | -0.001 | 0 |
| **DHEAS | 0.036 | 0.012 | 2.894 | 0.004 | 0.012 | 0.06 |
| Testosterone | 0.012 | 0.007 | 1.814 | 0.07 | -0.001 | 0.026 |
| CRP | 0.001 | 0.004 | 0.294 | 0.769 | -0.007 | 0.01 |
| Sleep_quality | 0.039 | 0.043 | 0.912 | 0.362 | -0.045 | 0.124 |
| Sampling_time | -0.002 | 0.027 | -0.063 | 0.95 | -0.054 | 0.051 |
| BMI | -0.001 | 0.006 | -0.178 | 0.859 | -0.013 | 0.011 |
| *Age | 0.007 | 0.003 | 2.487 | 0.013 | 0.002 | 0.013 |
| Income | 0.006 | 0.012 | 0.51 | 0.61 | -0.018 | 0.031 |
| Education | 0.001 | 0.011 | 0.103 | 0.918 | -0.02 | 0.023 |
| Feel_unhealthy | -0.019 | 0.037 | -0.527 | 0.598 | -0.092 | 0.053 |
| Diseases | -0.023 | 0.015 | -1.492 | 0.136 | -0.053 | 0.007 |
| Sport | -0.015 | 0.016 | -0.923 | 0.356 | -0.046 | 0.017 |
| Fruits_vegetables | 0.001 | 0.029 | 0.051 | 0.959 | -0.055 | 0.058 |
| Alcohol_amount | 0.018 | 0.02 | 0.926 | 0.354 | -0.02 | 0.056 |
| **Alcohol_frequency | -0.05 | 0.019 | -2.652 | 0.008 | -0.087 | -0.013 |
| Smoking | -0.025 | 0.018 | -1.417 | 0.157 | -0.061 | 0.01 |
| Children | -0.034 | 0.045 | -0.772 | 0.44 | -0.122 | 0.053 |
| *Intercept* | 2.178 | 0.482 | 4.515 | < 0.0001 | 1.233 | 3.124 |
| Household_ID *Var* | 0.092 | 0.084 |  |  |  |  |

Household code was modeled as a random (group) effect (*Var*), variables affecting biomarker level as fixed effects. For details on the rest of the variables, see Supplemental Table S1. Binary categorical variables (partner, sex) have the reference category designated with T. Asterisks denote the significant associations. * p<0.05, ** p<0.01, *** p<0.001, **** p<0.0001, chi-square test.

Tab. S2C: Mixed effects linear regression model of total high-density lipoprotein (HDL) cholesterol.

| *Dependent variable:* | ***HDL*** | *Log-Likelihood:* | -280.96 |  |  |  |
| --- | --- | --- | --- | --- | --- | --- |
| *No. Groups:* | 944 | *Model:* | MixedLM |  |  |  |
| *Min. group size:* | 1 | *Scale:* | 0.0801 |  |  |  |
| *Max. group size:* | 3 | *Observations:* | 944 |  |  |  |
| *Mean group size:* | 1.2 | *Converged:* | Yes |  |  |  |
| ***Variable*** | ***Coef.*** | ***Std.Err.*** | ***z*** | ***P>\|z\|*** | ***CI (0.025)*** | ***CI (0.975)*** |
| **Social_jetlag** | **0.014** | 0.015 | 0.963 | **0.336** | -0.015 | 0.043 |
| ***Chronotype** | **-0.023** | 0.011 | -2.159 | **0.031** | -0.045 | -0.002 |
| ****Sex[T.women] | 0.336 | 0.043 | 7.746 | < 0.0001 | 0.251 | 0.421 |
| *Partner[T.Yes] | 0.06 | 0.026 | 2.326 | 0.02 | 0.009 | 0.111 |
| *LDL | 0.023 | 0.011 | 2.052 | 0.04 | 0.001 | 0.044 |
| ****TAG | -0.124 | 0.014 | -9.189 | < 0.0001 | -0.151 | -0.098 |
| Glucose | -0.014 | 0.008 | -1.675 | 0.094 | -0.031 | 0.002 |
| ****Cortisol | 0 | 0 | 4.513 | < 0.0001 | 0 | 0 |
| DHEAS | -0.006 | 0.005 | -1.411 | 0.158 | -0.015 | 0.002 |
| Testosterone | 0.002 | 0.002 | 0.764 | 0.445 | -0.003 | 0.007 |
| CRP | 0.002 | 0.002 | 1.123 | 0.261 | -0.001 | 0.005 |
| Sleep_quality | 0.001 | 0.016 | 0.051 | 0.959 | -0.03 | 0.032 |
| Sampling_time | 0.008 | 0.01 | 0.837 | 0.402 | -0.011 | 0.027 |
| ****BMI | -0.017 | 0.002 | -7.679 | < 0.0001 | -0.021 | -0.013 |
| Age | 0 | 0.001 | -0.336 | 0.737 | -0.002 | 0.002 |
| Income | -0.002 | 0.005 | -0.391 | 0.695 | -0.011 | 0.007 |
| Education | 0.006 | 0.004 | 1.477 | 0.14 | -0.002 | 0.014 |
| Feel_unhealthy | 0.02 | 0.013 | 1.467 | 0.142 | -0.007 | 0.046 |
| Diseases | -0.01 | 0.006 | -1.726 | 0.084 | -0.021 | 0.001 |
| Sport | -0.002 | 0.006 | -0.293 | 0.769 | -0.013 | 0.01 |
| Fruits_vegetables | 0.008 | 0.01 | 0.726 | 0.468 | -0.013 | 0.028 |
| Alcohol_amount | 0.003 | 0.007 | 0.404 | 0.687 | -0.011 | 0.017 |
| ****Alcohol_freqnc. | 0.039 | 0.007 | 5.787 | < 0.0001 | 0.026 | 0.053 |
| *Smoking | -0.016 | 0.007 | -2.404 | 0.016 | -0.028 | -0.003 |
| **Children | -0.055 | 0.016 | -3.355 | 0.001 | -0.086 | -0.023 |
| *Intercept* | 1.59 | 0.171 | 9.318 | < 0.0001 | 1.256 | 1.925 |
| Household_ID *Var* | 0.018 | 0.043 |  |  |  |  |

Household code was modeled as a random (group) effect (*Var*), variables affecting biomarker level as fixed effects. For details on the rest of the variables, see Supplemental Table S1. Binary categorical variables (partner, sex) have the reference category designated with T. Asterisks denote the significant associations. * p<0.05, ** p<0.01, *** p<0.001, **** p<0.0001, chi-square test.

Tab. S2D: Mixed effects linear regression model of triglycerides.

| *Dependent variable:* | **triglycerides** | *Log-Likelihood:* | -1119.23 |  |  |  |
| --- | --- | --- | --- | --- | --- | --- |
| *No. Groups:* | 944 | *Model:* | MixedLM |  |  |  |
| *Min. group size:* | 1 | *Scale:* | 0.4257 |  |  |  |
| *Max. group size:* | 3 | *Observations:* | 1091 |  |  |  |
| *Mean group size:* | 1.2 | *Converged:* | Yes |  |  |  |
| ***Variable*** | ***Coef.*** | ***Std.Err.*** | ***z*** | ***P>\|z\|*** | ***CI (0.025)*** | ***CI (0.975)*** |
| **Social_jetlag** | **-0.024** | 0.032 | -0.747 | **0.455** | -0.087 | 0.039 |
| **Chronotype** | **-0.032** | 0.023 | -1.362 | **0.173** | -0.078 | 0.014 |
| **Sex[T.women] | -0.323 | 0.096 | -3.352 | 0.001 | -0.511 | -0.134 |
| Partner[T.Yes] | -0.019 | 0.056 | -0.338 | 0.736 | -0.128 | 0.09 |
| ****LDL | 0.206 | 0.023 | 8.981 | < 0.0001 | 0.161 | 0.251 |
| ****HDL | -0.578 | 0.063 | -9.2 | < 0.0001 | -0.701 | -0.455 |
| **Glucose | 0.056 | 0.018 | 3.066 | 0.002 | 0.02 | 0.092 |
| ****Cortisol | 0.001 | 0 | 5.624 | < 0.0001 | 0.001 | 0.001 |
| **DHEAS | -0.026 | 0.01 | -2.632 | 0.008 | -0.045 | -0.007 |
| ****Testosterone | -0.019 | 0.005 | -3.49 | < 0.0001 | -0.029 | -0.008 |
| CRP | 0 | 0.003 | 0.059 | 0.953 | -0.006 | 0.007 |
| *Sleep_quality | -0.084 | 0.034 | -2.481 | 0.013 | -0.151 | -0.018 |
| Sampling_time | 0.003 | 0.021 | 0.152 | 0.879 | -0.038 | 0.044 |
| ****BMI | 0.024 | 0.005 | 5.055 | < 0.0001 | 0.015 | 0.034 |
| *Age | -0.005 | 0.002 | -2.148 | 0.032 | -0.009 | 0 |
| Income | -0.002 | 0.01 | -0.167 | 0.867 | -0.021 | 0.017 |
| Education | 0.004 | 0.009 | 0.491 | 0.623 | -0.013 | 0.021 |
| Feel_unhealthy | 0.012 | 0.029 | 0.416 | 0.678 | -0.045 | 0.069 |
| Diseases | 0.001 | 0.012 | 0.062 | 0.95 | -0.023 | 0.025 |
| Sport | 0.014 | 0.013 | 1.156 | 0.248 | -0.01 | 0.039 |
| Fruits_vegetables | -0.01 | 0.023 | -0.421 | 0.674 | -0.054 | 0.035 |
| Alcohol_amount | 0.016 | 0.015 | 1.022 | 0.307 | -0.014 | 0.046 |
| Alcohol_frequency | 0.026 | 0.015 | 1.775 | 0.076 | -0.003 | 0.055 |
| ****Smoking | 0.049 | 0.014 | 3.537 | < 0.0001 | 0.022 | 0.077 |
| Children | -0.048 | 0.035 | -1.374 | 0.169 | -0.116 | 0.02 |
| *Intercept* | 0.988 | 0.381 | 2.594 | 0.009 | 0.242 | 1.735 |
| Household_ID *Var* | 0.03 | 0.082 |  |  |  |  |

Household code was modeled as a random (group) effect (*Var*), variables affecting biomarker level as fixed effects. For details on the rest of the variables, see Supplemental Table S1. Binary categorical variables (partner, sex) have the reference category designated with T. Asterisks denote the significant associations. * p<0.05, ** p<0.01, *** p<0.001, **** p<0.0001, chi-square test.

Tab. S2E: Mixed effects linear regression model of glucose.

| *Dependent variable:* | ***glucose*** | *Log-Likelihood:* | -1667.91 |  |  |  |
| --- | --- | --- | --- | --- | --- | --- |
| *No. Groups:* | 944 | *Model:* | MixedLM |  |  |  |
| *Min. group size:* | 1 | *Scale:* | 1.0687 |  |  |  |
| *Max. group size:* | 3 | *Observations:* | 1091 |  |  |  |
| *Mean group size:* | 1.2 | *Converged:* | Yes |  |  |  |
| ***Variable*** | ***Coef.*** | ***Std.Err.*** | ***z*** | ***P>\|z\|*** | ***CI (0.025)*** | ***CI (0.975)*** |
| **Social_jetlag** | **0.041** | 0.053 | 0.778 | **0.437** | -0.063 | 0.145 |
| ****Chronotype** | **-0.114** | 0.038 | -2.952 | **0.003** | -0.189 | -0.038 |
| *Sex[T.women] | -0.361 | 0.159 | -2.272 | 0.023 | -0.672 | -0.05 |
| Partner[T.Yes] | -0.152 | 0.092 | -1.644 | 0.1 | -0.332 | 0.029 |
| LDL | -0.072 | 0.039 | -1.836 | 0.066 | -0.149 | 0.005 |
| HDL | -0.18 | 0.108 | -1.673 | 0.094 | -0.392 | 0.031 |
| **TAG | 0.155 | 0.05 | 3.111 | 0.002 | 0.057 | 0.253 |
| ****Cortisol | 0.001 | 0 | 4.44 | < 0.0001 | 0.001 | 0.002 |
| **DHEAS | 0.043 | 0.016 | 2.63 | 0.009 | 0.011 | 0.074 |
| Testosterone | -0.012 | 0.009 | -1.373 | 0.17 | -0.03 | 0.005 |
| CRP | 0.001 | 0.005 | 0.236 | 0.814 | -0.009 | 0.012 |
| Sleep_quality | 0.043 | 0.056 | 0.763 | 0.446 | -0.067 | 0.152 |
| Sampling_time | 0.005 | 0.035 | 0.135 | 0.893 | -0.063 | 0.073 |
| ****BMI | 0.03 | 0.008 | 3.741 | < 0.0001 | 0.014 | 0.046 |
| ****Age | 0.025 | 0.004 | 6.712 | < 0.0001 | 0.018 | 0.032 |
| Income | 0.028 | 0.016 | 1.754 | 0.079 | -0.003 | 0.06 |
| Education | -0.017 | 0.014 | -1.202 | 0.229 | -0.045 | 0.011 |
| *Feel_unhealthy | 0.11 | 0.048 | 2.287 | 0.022 | 0.016 | 0.203 |
| Diseases | 0.034 | 0.02 | 1.683 | 0.092 | -0.006 | 0.073 |
| *Sport | 0.041 | 0.021 | 1.967 | 0.049 | 0 | 0.081 |
| Fruits_vegetables | -0.054 | 0.037 | -1.453 | 0.146 | -0.127 | 0.019 |
| Alcohol_amount | 0.006 | 0.025 | 0.217 | 0.828 | -0.044 | 0.055 |
| Alcohol_frequency | 0.034 | 0.025 | 1.39 | 0.165 | -0.014 | 0.082 |
| **Smoking | 0.067 | 0.023 | 2.884 | 0.004 | 0.021 | 0.112 |
| Children | -0.004 | 0.058 | -0.065 | 0.948 | -0.117 | 0.11 |
| *Intercept* | 2.402 | 0.628 | 3.823 | < 0.0001 | 1.17 | 3.633 |
| Household_ID *Var* | 0.181 | 0.112 |  |  |  |  |

Household code was modeled as a random (group) effect (*Var*), variables affecting biomarker level as fixed effects. For details on the rest of the variables, see Supplemental Table S1. Binary categorical variables (partner, sex) have the reference category designated with T. Asterisks denote the significant associations. * p<0.05, ** p<0.01, *** p<0.001, **** p<0.0001, chi-square test.

Tab. S2F: Mixed effects linear regression model of cortisol.

| *Dependent variable:* | ***cortisol*** | *Log-Likelihood:* | -6841.28 |  |  |  |
| --- | --- | --- | --- | --- | --- | --- |
| *No. Groups:* | 944 | *Model:* | MixedLM |  |  |  |
| *Min. group size:* | 1 | *Scale:* | 15182.47 |  |  |  |
| *Max. group size:* | 3 | *Observations:* | 1091 |  |  |  |
| *Mean group size:* | 1.2 | *Converged:* | Yes |  |  |  |
| ***Variable*** | ***Coef.*** | ***Std.Err.*** | ***z*** | ***P>\|z\|*** | ***CI (0.025)*** | ***CI (0.975)*** |
| ***Social_jetlag** | **-13.506** | 6.062 | -2.228 | **0.026** | -25.387 | -1.624 |
| ******Chronotype** | **32.979** | 4.314 | 7.645 | **< 0.0001** | 24.524 | 41.433 |
| Sex[T.women] | 13.484 | 18.334 | 0.735 | 0.462 | -22.449 | 49.418 |
| Partner[T.Yes] | -3.027 | 10.541 | -0.287 | 0.774 | -23.688 | 17.633 |
| ****LDL | -17.307 | 4.476 | -3.867 | < 0.0001 | -26.08 | -8.535 |
| ****HDL | 54.934 | 12.26 | 4.481 | < 0.0001 | 30.905 | 78.963 |
| ****TAG | 31.838 | 5.661 | 5.624 | < 0.0001 | 20.742 | 42.934 |
| Glucose | 15.228 | 3.44 | 4.427 | < 0.0001 | 8.486 | 21.97 |
| DHEAS | 1.431 | 1.854 | 0.772 | 0.44 | -2.203 | 5.064 |
| Testosterone | 0.642 | 1.024 | 0.627 | 0.531 | -1.365 | 2.649 |
| **CRP | 1.676 | 0.626 | 2.679 | 0.007 | 0.45 | 2.902 |
| *Sleep_quality | 14.004 | 6.403 | 2.187 | 0.029 | 1.454 | 26.554 |
| ****Sampling_time | -45.422 | 3.708 | -12.251 | < 0.0001 | -52.689 | -38.155 |
| ****BMI | -4.475 | 0.911 | -4.91 | < 0.0001 | -6.261 | -2.689 |
| Age | 0.001 | 0.432 | 0.003 | 0.998 | -0.845 | 0.847 |
| Income | -0.977 | 1.832 | -0.533 | 0.594 | -4.568 | 2.614 |
| Education | -2.73 | 1.623 | -1.682 | 0.093 | -5.91 | 0.45 |
| *Feel_unhealthy | -11.545 | 5.489 | -2.103 | 0.035 | -22.303 | -0.787 |
| Diseases | 4.025 | 2.296 | 1.753 | 0.08 | -0.475 | 8.525 |
| Sport | 1.416 | 2.373 | 0.597 | 0.551 | -3.236 | 6.067 |
| Fruits_vegetables | -2.117 | 4.285 | -0.494 | 0.621 | -10.516 | 6.281 |
| Alcohol_amount | -1.187 | 2.911 | -0.408 | 0.684 | -6.892 | 4.519 |
| Alcohol_frequency | 0.787 | 2.811 | 0.28 | 0.779 | -4.722 | 6.296 |
| *Smoking | -5.591 | 2.66 | -2.102 | 0.036 | -10.803 | -0.378 |
| Children | 2.074 | 6.604 | 0.314 | 0.753 | -10.869 | 15.017 |
| *Intercept* | 666.889 | 69.566 | 9.586 | < 0.0001 | 530.541 | 803.236 |
| Household_ID *Var* | 1204.193 | 11.549 |  |  |  |  |

Household code was modeled as a random (group) effect (*Var*), variables affecting biomarker level as fixed effects. For details on the rest of the variables, see Supplemental Table S1. Binary categorical variables (partner, sex) have the reference category designated with T. Asterisks denote the significant associations. * p<0.05, ** p<0.01, *** p<0.001, **** p<0.0001, chi-square test.

Tab. S2G: Mixed effects linear regression model of testosterone.

| *Dependent variable:* | ***testosterone*** | *Log-Likelihood:* | -3004.78 |  |  |  |
| --- | --- | --- | --- | --- | --- | --- |
| *No. Groups:* | 944 | *Model:* | MixedLM |  |  |  |
| *Min. group size:* | 1 | *Scale:* | 13.75 |  |  |  |
| *Max. group size:* | 3 | *Observations:* | 1091 |  |  |  |
| *Mean group size:* | 1.2 | *Converged:* | Yes |  |  |  |
| ***Variable*** | ***Coef.*** | ***Std.Err.*** | ***z*** | ***P>\|z\|*** | ***CI (0.025)*** | ***CI (0.975)*** |
| **Social_jetlag** | **-0.219** | 0.18 | -1.215 | **0.224** | -0.572 | 0.134 |
| **Chronotype** | **0.054** | 0.132 | 0.408 | **0.684** | -0.204 | 0.311 |
| ****Sex[T.women] | -14.984 | 0.299 | -50.061 | < 0.0001 | -15.57 | -14.397 |
| Partner[T.Yes] | 0.233 | 0.312 | 0.746 | 0.456 | -0.379 | 0.845 |
| LDL | 0.259 | 0.134 | 1.93 | 0.054 | -0.004 | 0.522 |
| HDL | 0.29 | 0.367 | 0.791 | 0.429 | -0.429 | 1.01 |
| ****TAG | -0.591 | 0.17 | -3.485 | < 0.0001 | -0.924 | -0.259 |
| Cortisol | 0.001 | 0.001 | 0.659 | 0.51 | -0.001 | 0.002 |
| DHEAS | -0.078 | 0.055 | -1.417 | 0.157 | -0.186 | 0.03 |
| Glucose | -0.147 | 0.103 | -1.426 | 0.154 | -0.349 | 0.055 |
| CRP | -0.007 | 0.019 | -0.356 | 0.722 | -0.043 | 0.03 |
| Sleep_quality | 0.286 | 0.19 | 1.504 | 0.133 | -0.087 | 0.66 |
| Sampling_time | 0.001 | 0.117 | 0.011 | 0.991 | -0.229 | 0.231 |
| **BMI | -0.072 | 0.027 | -2.634 | 0.008 | -0.125 | -0.018 |
| Age | -0.012 | 0.013 | -0.95 | 0.342 | -0.037 | 0.013 |
| Income | -0.012 | 0.054 | -0.223 | 0.824 | -0.119 | 0.094 |
| Education | -0.043 | 0.048 | -0.892 | 0.373 | -0.137 | 0.051 |
| Feel_unhealthy | -0.1 | 0.163 | -0.612 | 0.541 | -0.42 | 0.22 |
| Diseases | -0.034 | 0.068 | -0.498 | 0.618 | -0.168 | 0.1 |
| Sport | -0.105 | 0.07 | -1.495 | 0.135 | -0.243 | 0.033 |
| Fruits_vegetables | -0.012 | 0.127 | -0.097 | 0.923 | -0.262 | 0.237 |
| Alcohol_amount | -0.139 | 0.086 | -1.612 | 0.107 | -0.308 | 0.03 |
| *Alcohol_frequency | -0.203 | 0.083 | -2.442 | 0.015 | -0.365 | -0.04 |
| Smoking | 0.126 | 0.079 | 1.598 | 0.11 | -0.029 | 0.281 |
| Children | -0.258 | 0.196 | -1.319 | 0.187 | -0.641 | 0.125 |
| *Intercept* | 20.234 | 2.062 | 9.815 | < 0.0001 | 16.194 | 24.275 |
| Household_ID *Var* | 0.701 | 0.244 |  |  |  |  |

Household code was modeled as a random (group) effect (*Var*), variables affecting biomarker level as fixed effects. For details on the rest of the variables, see Supplemental Table S1. Binary categorical variables (partner, sex) have the reference category designated with T. Asterisks denote the significant associations. * p<0.05, ** p<0.01, *** p<0.001, **** p<0.0001, chi-square test.

Tab. S2H: Mixed effects linear regression model of dehydroepiandrosterone (DHEAS).

| *Dependent variable:* | ***DHEAS*** | *Log-Likelihood:* | -2351.4 |  |  |  |
| --- | --- | --- | --- | --- | --- | --- |
| *No. Groups:* | 944 | *Model:* | MixedLM |  |  |  |
| *Min. group size:* | 1 | *Scale:* | 4.2994 |  |  |  |
| *Max. group size:* | 3 | *Observations:* | 1091 |  |  |  |
| *Mean group size:* | 1.2 | *Converged:* | Yes |  |  |  |
| ***Variable*** | ***Coef.*** | ***Std.Err.*** | ***z*** | ***P>\|z\|*** | ***CI (0.025)*** | ***CI (0.975)*** |
| **Social_jetlag** | **-0.109** | 0.099 | -1.097 | **0.272** | -0.304 | 0.086 |
| **Chronotype** | **0.064** | 0.072 | 0.881 | **0.378** | -0.078 | 0.205 |
| ****Sex[T.women] | -1.737 | 0.294 | -5.906 | < 0.0001 | -2.314 | -1.161 |
| Partner[T.Yes] | -0.376 | 0.171 | -2.196 | 0.028 | -0.711 | -0.04 |
| **LDL | 0.206 | 0.073 | 2.807 | 0.005 | 0.062 | 0.35 |
| HDL | -0.262 | 0.202 | -1.299 | 0.194 | -0.657 | 0.133 |
| **TAG | -0.245 | 0.093 | -2.619 | 0.009 | -0.428 | -0.062 |
| Cortisol | 0 | 0 | 0.775 | 0.438 | -0.001 | 0.001 |
| *Glucose | 0.141 | 0.057 | 2.494 | 0.013 | 0.03 | 0.252 |
| Testosterone | -0.024 | 0.017 | -1.423 | 0.155 | -0.056 | 0.009 |
| CRP | -0.008 | 0.01 | -0.759 | 0.448 | -0.028 | 0.012 |
| Sleep_quality | 0.08 | 0.105 | 0.763 | 0.445 | -0.125 | 0.285 |
| Sampling_time | -0.002 | 0.064 | -0.034 | 0.973 | -0.128 | 0.124 |
| BMI | -0.015 | 0.015 | -0.994 | 0.32 | -0.044 | 0.015 |
| ****Age | -0.1 | 0.006 | -15.769 | < 0.0001 | -0.112 | -0.087 |
| Income | 0 | 0.03 | -0.004 | 0.997 | -0.058 | 0.058 |
| Education | 0.021 | 0.026 | 0.801 | 0.423 | -0.031 | 0.073 |
| *Feel_unhealthy | -0.184 | 0.09 | -2.057 | 0.04 | -0.36 | -0.009 |
| Diseases | 0.044 | 0.038 | 1.16 | 0.246 | -0.03 | 0.117 |
| Sport | -0.013 | 0.039 | -0.334 | 0.738 | -0.089 | 0.063 |
| Fruits_vegetables | 0.019 | 0.07 | 0.27 | 0.787 | -0.118 | 0.156 |
| Alcohol_amount | 0.041 | 0.047 | 0.871 | 0.384 | -0.051 | 0.134 |
| Alcohol_frequency | 0.012 | 0.046 | 0.266 | 0.79 | -0.077 | 0.102 |
| Smoking | 0.081 | 0.043 | 1.876 | 0.061 | -0.004 | 0.166 |
| Children | 0.057 | 0.107 | 0.529 | 0.597 | -0.153 | 0.266 |
| *Intercept* | 10.201 | 1.14 | 8.95 | < 0.0001 | 7.967 | 12.435 |
| Household_ID *Var* | 0.062 | 0.134 |  |  |  |  |

Household code was modeled as a random (group) effect (*Var*), variables affecting biomarker level as fixed effects. For details on the rest of the variables, see Supplemental Table S1. Binary categorical variables (partner, sex) have the reference category designated with T. Asterisks denote the significant associations. * p<0.05, ** p<0.01, *** p<0.001, **** p<0.0001, chi-square test.

Tab. S2I: Mixed effects linear regression model of C-reactive protein (CRP).

| *Dependent variable:* | ***CRP*** | *Log-Likelihood:* | -3533.64 |  |  |  |
| --- | --- | --- | --- | --- | --- | --- |
| *No. Groups:* | 944 | *Model:* | MixedLM |  |  |  |
| *Min. group size:* | 1 | *Scale:* | 38.09 |  |  |  |
| *Max. group size:* | 3 | *Observations:* | 1091 |  |  |  |
| *Mean group size:* | 1.2 | *Converged:* | Yes |  |  |  |
| ***Variable*** | ***Coef.*** | ***Std.Err.*** | ***z*** | ***P>\|z\|*** | ***CI (0.025)*** | ***CI (0.975)*** |
| **Social_jetlag** | **-0.049** | 0.293 | -0.169 | **0.866** | -0.623 | 0.525 |
| ***Chronotype** | **0.513** | 0.213 | 2.408 | **0.016** | 0.095 | 0.931 |
| Sex[T.women] | -0.035 | 0.884 | -0.04 | 0.968 | -1.769 | 1.698 |
| Partner[T.Yes] | -0.635 | 0.506 | -1.257 | 0.209 | -1.626 | 0.356 |
| LDL | 0.086 | 0.218 | 0.394 | 0.694 | -0.341 | 0.513 |
| HDL | 0.526 | 0.602 | 0.873 | 0.383 | -0.655 | 1.706 |
| TAG | 0.009 | 0.277 | 0.033 | 0.974 | -0.534 | 0.552 |
| **Cortisol | 0.004 | 0.001 | 2.711 | 0.007 | 0.001 | 0.007 |
| DHEAS | -0.068 | 0.089 | -0.762 | 0.446 | -0.244 | 0.107 |
| Testosterone | -0.017 | 0.049 | -0.34 | 0.734 | -0.113 | 0.08 |
| Glucose | 0.041 | 0.167 | 0.245 | 0.806 | -0.287 | 0.369 |
| *Sleep_quality | -0.707 | 0.309 | -2.291 | 0.022 | -1.312 | -0.102 |
| Sampling_time | 0.16 | 0.19 | 0.842 | 0.4 | -0.212 | 0.532 |
| ****BMI | 0.259 | 0.044 | 5.911 | < 0.0001 | 0.173 | 0.346 |
| Age | -0.036 | 0.021 | -1.741 | 0.082 | -0.077 | 0.005 |
| Income | 0.147 | 0.088 | 1.667 | 0.096 | -0.026 | 0.319 |
| Education | -0.014 | 0.078 | -0.176 | 0.86 | -0.167 | 0.14 |
| Feel_unhealthy | 0.329 | 0.265 | 1.244 | 0.213 | -0.189 | 0.848 |
| Diseases | 0.179 | 0.111 | 1.621 | 0.105 | -0.038 | 0.396 |
| Sport | -0.01 | 0.115 | -0.087 | 0.931 | -0.235 | 0.215 |
| Fruits_vegetables | -0.337 | 0.206 | -1.634 | 0.102 | -0.74 | 0.067 |
| Alcohol_amount | -0.16 | 0.14 | -1.143 | 0.253 | -0.434 | 0.114 |
| Alcohol_frequency | -0.011 | 0.135 | -0.081 | 0.936 | -0.275 | 0.254 |
| Smoking | 0.242 | 0.128 | 1.891 | 0.059 | -0.009 | 0.493 |
| Children | -0.22 | 0.316 | -0.695 | 0.487 | -0.839 | 0.4 |
| *Intercept* | -5.371 | 3.485 | -1.541 | 0.123 | -12.2 | 1.46 |
| Household_ID *Var* | 0 | 0.692 |  |  |  |  |

Household code was modeled as a random (group) effect (*Var*), variables affecting biomarker level as fixed effects. For details on the rest of the variables, see Supplemental Table S1. Binary categorical variables (partner, sex) have the reference category designated with T. Asterisks denote the significant associations. * p<0.05, ** p<0.01, *** p<0.001, **** p<0.0001, chi-square test.

Tab. S2J: Mixed effects linear regression model of atherogenic index of plasma (AIP).

| *Dependent variable:* | ***AIP*** | *Log-Likelihood:* | -855.04 |  |  |  |
| --- | --- | --- | --- | --- | --- | --- |
| *No. Groups:* | 944 | *Model:* | MixedLM |  |  |  |
| *Min. group size:* | 1 | *Scale:* | 0.2333 |  |  |  |
| *Max. group size:* | 3 | *Observations:* | 1091 |  |  |  |
| *Mean group size:* | 1.2 | *Converged:* | Yes |  |  |  |
| ***Variable*** | ***Coef.*** | ***Std.Err.*** | ***z*** | ***P>\|z\|*** | ***CI (0.025)*** | ***CI (0.975)*** |
| **Social_jetlag** | **-0.038** | 0.025 | -1.528 | **0.127** | -0.088 | 0.011 |
| **Chronotype** | **0.015** | 0.018 | 0.813 | **0.416** | -0.021 | 0.051 |
| ****Sex[T.women] | -0.638 | 0.072 | -8.827 | < 0.0001 | -0.78 | -0.497 |
| Partner[T.Yes] | -0.083 | 0.044 | -1.899 | 0.058 | -0.169 | 0.003 |
| ****LDL | 0.153 | 0.018 | 8.489 | < 0.0001 | 0.118 | 0.188 |
| ****Glucose | 0.059 | 0.014 | 4.109 | < 0.0001 | 0.031 | 0.087 |
| **Cortisol | 0 | 0 | 2.783 | 0.005 | 0 | 0.001 |
| DHEAS | -0.014 | 0.008 | -1.851 | 0.064 | -0.029 | 0.001 |
| ****Testosterone | -0.017 | 0.004 | -3.982 | < 0.0001 | -0.025 | -0.008 |
| CRP | -0.001 | 0.003 | -0.539 | 0.59 | -0.006 | 0.004 |
| *Sleep_quality | -0.058 | 0.027 | -2.196 | 0.028 | -0.111 | -0.006 |
| Sampling_time | -0.005 | 0.016 | -0.28 | 0.779 | -0.037 | 0.028 |
| ****BMI | 0.04 | 0.004 | 11.078 | < 0.0001 | 0.033 | 0.047 |
| Age | -0.003 | 0.002 | -1.561 | 0.118 | -0.006 | 0.001 |
| Income | 0.001 | 0.008 | 0.149 | 0.882 | -0.014 | 0.016 |
| Education | -0.003 | 0.007 | -0.428 | 0.669 | -0.016 | 0.01 |
| Feel_unhealthy | -0.003 | 0.023 | -0.149 | 0.881 | -0.048 | 0.041 |
| Diseases | 0.013 | 0.01 | 1.39 | 0.164 | -0.005 | 0.032 |
| Sport | 0.012 | 0.01 | 1.26 | 0.208 | -0.007 | 0.032 |
| Fruits_vegetables | -0.013 | 0.018 | -0.726 | 0.468 | -0.048 | 0.022 |
| Alcohol_amount | 0.011 | 0.012 | 0.899 | 0.368 | -0.013 | 0.035 |
| *Alcohol_frequency | -0.023 | 0.012 | -1.992 | 0.046 | -0.045 | 0 |
| ****Smoking | 0.055 | 0.011 | 5.033 | < 0.0001 | 0.034 | 0.076 |
| Children | 0.007 | 0.027 | 0.263 | 0.792 | -0.047 | 0.061 |
| *Intercept* | -1.321 | 0.289 | -4.572 | < 0.0001 | -1.887 | -0.755 |
| Household_ID *Var* | 0.049 | 0.063 |  |  |  |  |

Household code was modeled as a random (group) effect (*Var*), variables affecting biomarker level as fixed effects. For details on the rest of the variables, see Supplemental Table S1. Binary categorical variables (partner, sex) have the reference category designated with T. Asterisks denote the significant associations. * p<0.05, ** p<0.01, *** p<0.001, **** p<0.0001, chi-square test.

Tab. S3: Simplified mixed linear regression model of total cholesterol.

| *Dependent variable:* | ***Cholesterol*** | *Log-Likelihood:* | -1639 |  |  |  |
| --- | --- | --- | --- | --- | --- | --- |
| *No. Groups:* | 1038 | *Model:* | MixedLM |  |  |  |
| *Min. group size:* | 1 | *Scale:* | 0. 7268 |  |  |  |
| *Max. group size:* | 3 | *Observations:* | 1218 |  |  |  |
| *Mean group size:* | 1.2 | *Converged:* | Yes |  |  |  |
| ***Variable*** | ***Coef.*** | ***Std.Err.*** | ***z*** | ***P>\|z\|*** | ***CI (0.025)*** | ***CI (0.975)*** |
| ****Social_jetlag** | **0.13** | 0.04 | 3.287 | **0.001** | 0.053 | 0.208 |
| **Chronotype** | **-0.025** | 0.028 | -0.899 | **0.369** | -0.081 | 0.03 |
| **** Sex[T.women] | 0.451 | 0.054 | 8.331 | 7.99E-17 | 0.345 | 0.557 |
| ****TAG | 0.45 | 0.035 | 12.902 | 4.37E-38 | 0.381 | 0.518 |
| ****BMI | -0.027 | 0.006 | -4.796 | 1.62E-06 | -0.038 | -0.016 |
| **Age | 0.006 | 0.002 | 2.942 | 0.003 | 0.002 | 0.01 |
| *Diseases | -0.032 | 0.014 | -2.301 | 0.021 | -0.059 | -0.005 |
| *Intercept* | 4.603 | 0.209 | 22.062 | 7.3E-107 | 4.194 | 5.012 |
| Household_ID *Var* | 0.141 | 0.097 |  |  |  |  |

Household code was modeled as a random (group) effect (*Var*), variables affecting Cholesterol level as fixed effects (exposures). Binary categorical variable (sex) has the reference category designated with T. For details on the rest of the variables, see Supplemental Table S1. TAG – triglycerides, BMI – body-mas index, * p<0.05, ** p<0.01, **** p<0.0001, chi-square test.

Tab. S4: Simplified mixed linear regression model of total LDL cholesterol.

| *Dependent variable:* | ***LDL*** | *Log-Likelihood:* | -1575 |  |  |  |
| --- | --- | --- | --- | --- | --- | --- |
| *No. Groups:* | 1036 | *Model:* | MixedLM |  |  |  |
| *Min. group size:* | 1 | *Scale:* | 0. 7083 |  |  |  |
| *Max. group size:* | 3 | *Observations:* | 1215 |  |  |  |
| *Mean group size:* | 1.2 | *Converged:* | Yes |  |  |  |
| ***Variable*** | ***Coef.*** | ***Std.Err.*** | ***z*** | ***P>\|z\|*** | ***CI (0.025)*** | ***CI (0.975)*** |
| ****Social_jetlag** | **0.109** | 0.038 | 2.88 | **0.004** | 0.035 | 0.183 |
| **Chronotype** | **-0.018** | 0.027 | -0.667 | **0.505** | -0.071 | 0.035 |
| *Sex[T.women] | 0.118 | 0.057 | 2.079 | 0.038 | 0.007 | 0.229 |
| ****TAG | 0.27 | 0.035 | 7.747 | 9.44E-15 | 0.202 | 0.339 |
| *HDL | 0.16 | 0.079 | 2.016 | 0.044 | 0.004 | 0.315 |
| BMI | -0.001 | 0.006 | -0.14 | 0.889 | -0.012 | 0.01 |
| Age | 0.002 | 0.002 | 1.096 | 0.273 | -0.002 | 0.006 |
| *Diseases | -0.027 | 0.013 | -2.038 | 0.042 | -0.053 | -0.001 |
| *Intercept* | 2.353 | 0.249 | 9.463 | 2.99E-21 | 1.865 | 2.84 |
| Household_ID *Var* | 0.075 | 0.076 |  |  |  |  |

Household code was modeled as a random (group) effect (*Var*), variables affecting LDL level as fixed effects. Binary categorical variable (sex) has the reference category designated with T. For details on the rest of the variables, see Supplemental Table S1. TAG – triglycerides, BMI – body-mass index, * p<0.05, ** p<0.01, **** p<0.0001, chi-square test.

Tab. S5: Simplified mixed linear regression model of HDL.

| *Dependent variable:* | ***HDL*** | *Log-Likelihood:* | -338 |  |  |  |
| --- | --- | --- | --- | --- | --- | --- |
| *No. Groups:* | 1036 | *Model:* | MixedLM |  |  |  |
| *Min. group size:* | 1 | *Scale:* | 0. 0763 |  |  |  |
| *Max. group size:* | 3 | *Observations:* | 1216 |  |  |  |
| *Mean group size:* | 1.2 | *Converged:* | Yes |  |  |  |
| ***Variable*** | ***Coef.*** | ***Std.Err.*** | ***z*** | ***P>\|z\|*** | ***CI (0.025)*** | ***CI (0.975)*** |
| *MAD_ MSF_sasc_ | -0.032 | 0.015 | -2.176 | 0.03 | -0.06 | -0.003 |
| Social_jetlag | 0.01 | 0.013 | 0.741 | 0.459 | -0.016 | 0.036 |
| ****Sex[T.women] | 0.279 | 0.019 | 15.069 | 2.60E-51 | 0.243 | 0.315 |
| LDL | 0.02 | 0.01 | 1.885 | 0.059 | -0.001 | 0.04 |
| ****TAG | -0.117 | 0.012 | -9.429 | 4.12E-21 | -0.142 | -0.093 |
| ****BMI | -0.02 | 0.002 | -10.248 | 1.21E-24 | -0.024 | -0.016 |
| *Age | 0.002 | 0.001 | 2.518 | 0.012 | 0 | 0.003 |
| Diseases | -0.005 | 0.005 | -1.043 | 0.297 | -0.014 | 0.004 |
| *Intercept* | 1.864 | 0.071 | 26.313 | 1.3E-152 | 1.725 | 2.002 |
| Household_ID *Var* | 0.027 | 0.04 |  |  |  |  |

Household code was modeled as a random (group) effect (*Var*), variables affecting HDL level as fixed effects. Binary categorical variable (sex) has the reference category designated with T. For details on the rest of the variables, see Supplemental Table S1. TAG – triglycerides, BMI – body-mass index, MAD_MSF_sasc_ – median absolute deviation of normalized chronotype MSF_sasc_, * p<0.05, **** p<0.0001, chi-square test.

Tab. S6: Simplified mixed linear regression model of triglycerides.

| *Dependent variable:* | ***TAG*** | *Log-Likelihood:* | -1305 |  |  |  |
| --- | --- | --- | --- | --- | --- | --- |
| *No. Groups:* | 1036 | *Model:* | MixedLM |  |  |  |
| *Min. group size:* | 1 | *Scale:* | 0. 4690 |  |  |  |
| *Max. group size:* | 3 | *Observations:* | 1216 |  |  |  |
| *Mean group size:* | 1.2 | *Converged:* | Yes |  |  |  |
| ***Variable*** | ***Coef.*** | ***Std.Err.*** | ***z*** | ***P>\|z\|*** | ***CI (0.025)*** | ***CI (0.975)*** |
| ****MAD_ MSF_sasc_** | **0.085** | 0.032 | 2.62 | **0.009** | 0.021 | 0.148 |
| **Social_jetlag** | **-0.018** | 0.03 | -0.603 | **0.546** | -0.076 | 0.04 |
| Sex[T.women] | -0.06 | 0.045 | -1.317 | 0.188 | -0.149 | 0.029 |
| ****LDL | 0.174 | 0.022 | 7.752 | 9.05E-15 | 0.13 | 0.218 |
| ****HDL | -0.576 | 0.061 | -9.4 | 5.48E-21 | -0.696 | -0.456 |
| ****BMI | 0.028 | 0.004 | 6.323 | 2.57E-10 | 0.019 | 0.037 |
| Age | 0 | 0.002 | 0.27 | 0.787 | -0.003 | 0.003 |
| Diseases | 0.014 | 0.011 | 1.316 | 0.188 | -0.007 | 0.035 |
| *Intercept* | 0.875 | 0.194 | 4.505 | 6.63E-06 | 0.494 | 1.256 |
| Household_ID *Var* | 0.032 | 0.065 |  |  |  |  |

Household code was modeled as a random (group) effect (*Var*), variables affecting TAG level as fixed effects. Binary categorical variable (sex) is designated with C and reference category with T. For details on the rest of the variables, see Supplemental Table S1. TAG – triglycerides, BMI – body-mass index, MAD_MSF_sasc_ – median absolute deviation of normalized chronotype MSF_sasc_, * p<0.05, ** p<0.01, **** p<0.0001, chi-square test.

Tab. S7: Simplified mixed linear regression model of atherogenic index.

| *Dependent variable:* | ***AIP*** | *Log-Likelihood:* | -999 |  |  |  |
| --- | --- | --- | --- | --- | --- | --- |
| *No. Groups:* | 1036 | *Model:* | MixedLM |  |  |  |
| *Min. group size:* | 1 | *Scale:* | 0. 2522 |  |  |  |
| *Max. group size:* | 3 | *Observations:* | 1216 |  |  |  |
| *Mean group size:* | 1.2 | *Converged:* | Yes |  |  |  |
| ***Variable*** | ***Coef.*** | ***Std.Err.*** | ***z*** | ***P>\|z\|*** | ***CI (0.025)*** | ***CI (0.975)*** |
| ****MAD_MSF_sasc_** | **0.078** | 0.025 | 3.108 | **0.002** | 0.029 | 0.127 |
| **Social_jetlag** | **-0.021** | 0.023 | -0.926 | **0.354** | -0.067 | 0.024 |
| **** Sex[T.women] | -0.37 | 0.032 | -11.601 | 4.07E-31 | -0.432 | -0.307 |
| ****LDL | 0.128 | 0.017 | 7.345 | 2.05E-13 | 0.094 | 0.162 |
| ****BMI | 0.047 | 0.003 | 14.413 | 4.30E-47 | 0.04 | 0.053 |
| Age | -0.001 | 0.001 | -0.423 | 0.672 | -0.003 | 0.002 |
| *Diseases | 0.02 | 0.008 | 2.384 | 0.017 | 0.003 | 0.036 |
| *Intercept* | -1.625 | 0.122 | -13.322 | 1.72E-40 | -1.864 | -1.386 |
| Household_ID *Var* | 0.052 | 0.053 |  |  |  |  |

Household code was modeled as a random (group) effect (*Var*), variables affecting AIP as fixed effects. Binary categorical variable (sex) is designated has the reference category designated with T. For details on the rest of the variables, see Supplemental Table S1. AIP – atherogenic index of plasma, BMI – body-mass index, MAD_MSF_sasc_ – median absolute deviation of normalized chronotype MSF_sasc_, * p<0.05, ** p<0.01, **** p<0.0001, chi-square test.

Tab. S8: Mixed effects linear regression model of social jetlag components.

| *Dependent variable:* | ***social jetlag*** | *Log-Likelihood:* | -2829 |  |  |  |
| --- | --- | --- | --- | --- | --- | --- |
| *No. Groups:* | 1923 | *Model:* | MixedLM |  |  |  |
| *Min. group size:* | 1 | *Scale:* | 0.4049 |  |  |  |
| *Max. group size:* | 6 | *Observations:* | 2739 |  |  |  |
| *Mean group size:* | 1.4 | *Converged:* | Yes |  |  |  |
| ***Variable*** | ***Coef.*** | ***Std.Err.*** | ***z*** | ***P>\|z\|*** | ***CI (0.025)*** | ***CI (0.975)*** |
| Sex[T.women] | -0.032 | 0.029 | -1.124 | 0.261 | -0.088 | 0.024 |
| ** Partner [T.yes] | -0.099 | 0.035 | -2.855 | 0.004 | -0.167 | -0.031 |
| ****Chronotype | 0.186 | 0.015 | 12.783 | 2.04E-37 | 0.157 | 0.215 |
| ****Bamid | -0.023 | 0.005 | -4.991 | 6.02E-07 | -0.032 | -0.014 |
| ****Light_exposure | -0.017 | 0.004 | -3.946 | 7.95E-05 | -0.026 | -0.009 |
| ****Sleep_duration | -0.081 | 0.012 | -6.744 | 1.54E-11 | -0.105 | -0.057 |
| ****Sleep_quality | 0.095 | 0.021 | 4.486 | 7.27E-06 | 0.053 | 0.136 |
| ****Age | -0.014 | 0.001 | -12.127 | 7.63E-34 | -0.017 | -0.012 |
| ****Work | 0.005 | 0.001 | 5.723 | 1.05E-08 | 0.003 | 0.006 |
| **Commute_time | 0.003 | 0.001 | 3.075 | 0.002 | 0.001 | 0.004 |
| ****Income | 0.024 | 0.006 | 4.264 | 2.01E-05 | 0.013 | 0.035 |
| ****Education | -0.04 | 0.005 | -7.382 | 1.56E-13 | -0.051 | -0.029 |
| ****Time_stress | 0.029 | 0.004 | 7.561 | 3.99E-14 | 0.021 | 0.036 |
| Feel_rushed | 0.004 | 0.012 | 0.331 | 0.741 | -0.02 | 0.027 |
| Diseases | -0.012 | 0.008 | -1.44 | 0.15 | -0.028 | 0.004 |
| Feel_unhealthy | -0.02 | 0.018 | -1.147 | 0.251 | -0.055 | 0.015 |
| **Alcohol_amount | 0.029 | 0.009 | 3.132 | 0.002 | 0.011 | 0.047 |
| Alcohol_frequency | -0.002 | 0.009 | -0.261 | 0.794 | -0.02 | 0.016 |
| Smoking | 0.015 | 0.008 | 1.838 | 0.066 | -0.001 | 0.031 |
| Fruits_vegetables | 0.004 | 0.014 | 0.279 | 0.78 | -0.023 | 0.031 |
| Sport | -0.009 | 0.007 | -1.175 | 0.24 | -0.023 | 0.006 |
| *Children | -0.043 | 0.021 | -2.105 | 0.035 | -0.084 | -0.003 |
| *Settlement_size | -0.024 | 0.01 | -2.355 | 0.019 | -0.044 | -0.004 |
| *Latitude | 0.066 | 0.028 | 2.334 | 0.02 | 0.011 | 0.121 |
| **Longitude | 0.026 | 0.009 | 2.835 | 0.005 | 0.008 | 0.045 |
| *Intercept* | -2.083 | 1.473 | -1.414 | 0.157 | -4.97 | 0.804 |
| Household_ID *Var* | 0.06 | 0.026 |  |  |  |  |

Household code was modeled as a random (group) effect (*Var*), variables considered as affecting social jetlag size as fixed effects. Bamid (midpoint of perceived best alertness) was used as a proxy for subjective chronotype. Binary categorical variables (partner, sex) have the reference category designated with T. For details on the rest of the fixed effects variables, see Supplemental Table S1. Asterisks denote the significant associations. * p<0.05, ** p<0.01, **** p<0.0001, chi-square test.
